# MS_HistoneDB, a manually curated resource for proteomic analysis of human and mouse histones

**DOI:** 10.1186/s13072-016-0109-x

**Published:** 2017-01-10

**Authors:** Sara El Kennani, Annie Adrait, Alexey K. Shaytan, Saadi Khochbin, Christophe Bruley, Anna R. Panchenko, David Landsman, Delphine Pflieger, Jérôme Govin

**Affiliations:** 1 INSERM, U1038, CEA, BIG FR CNRS 3425-BGE, Université Grenoble Alpes, Grenoble, France; 2 National Center for Biotechnology Information, National Library of Medicine, National Institutes of Health, Bethesda, MD 20894 USA; 3 CNRS UMR 5309 INSERM U1209, Institute of Advanced Biosciences, Université Grenoble Alpes, Grenoble, France

**Keywords:** Histone, Histone variants, Chromatin, Mass spectrometry, Proteomics

## Abstract

**Background:**

Histones and histone variants are essential components of the nuclear chromatin. While mass spectrometry has opened a large window to their characterization and functional studies, their identification from proteomic data remains challenging. Indeed, the current interpretation of mass spectrometry data relies on public databases which are either not exhaustive (Swiss-Prot) or contain many redundant entries (UniProtKB or NCBI). Currently, no protein database is ideally suited for the analysis of histones and the complex array of mammalian histone variants.

**Results:**

We propose two proteomics-oriented manually curated databases for mouse and human histone variants. We manually curated >1700 gene, transcript and protein entries to produce a non-redundant list of 83 mouse and 85 human histones. These entries were annotated in accordance with the current nomenclature and unified with the “HistoneDB2.0 with Variants” database. This resource is provided in a format that can be directly read by programs used for mass spectrometry data interpretation. In addition, it was used to interpret mass spectrometry data acquired on histones extracted from mouse testis. Several histone variants, which had so far only been inferred by homology or detected at the RNA level, were detected by mass spectrometry, confirming the existence of their protein form.

**Conclusions:**

Mouse and human histone entries were collected from different databases and subsequently curated to produce a non-redundant protein-centric resource, MS_HistoneDB. It is dedicated to the proteomic study of histones in mouse and human and will hopefully facilitate the identification and functional study of histone variants.

**Electronic supplementary material:**

The online version of this article (doi:10.1186/s13072-016-0109-x) contains supplementary material, which is available to authorized users.

## Background

In eukaryotic cells, the nucleosome is the basic unit of chromatin organization. Nucleosomes are composed of an octamer of four core histones, H2A, H2B, H3 and H4, wrapped by DNA [1]. An additional linker histone, H1, can be deposited near the DNA entry–exit points [2, 3]. The dynamic organization of chromatin impacts many cellular events, including the regulation of gene transcription, DNA replication and the maintenance of genome integrity through DNA repair mechanisms [4, 5]. These pathways signal to chromatin by different mechanisms including DNA methylation, non-coding regulatory RNAs, recruitment of remodelling factors, incorporation of histone variants and covalent modifications of histones [6–12]. Histones are decorated by many post-translational modifications, the most common of which are acetylation, methylation, phosphorylation and ubiquitination [13, 14]. Some of these modifications favour transcription activation, while others are associated with repression of transcription [15]. In addition to transcription, histone modifications are involved in numerous regulatory circuits, such as chromosome dynamics [16], DNA repair [17] or the establishment and maintenance of heterochromatin [18]. Furthermore, dedicated molecular machineries can load and mobilize nucleosomes along the DNA (for review, see [4]). These chromatin remodellers play an important role in the regulation of transcription by organizing the nucleosomal positions at critical regulatory regions [19, 20]. Finally, non-allelic variants of canonical histones, named histone variants, are important elements in chromatin signalling pathways [21, 22]. Some variants are general players—expressed ubiquitously, contributing to various aspects of transcription and epigenetic regulations—while others are only expressed in certain cell types, such as germ cells [23]. Some of these variants are specifically expressed during sperm differentiation and are annotated TS for testis-specific [24–26]. Altogether, histone variants have been described for H3, H2A, H2B and H1; H4 is the only histone for which no variant has been identified in mammals, but some organisms, such as the urochordate *Oikopleura dioica*, ciliates and trypanosomes, have evolved H4 variants [10, 27–29].

Histone variants were initially discovered using classical biochemical approaches. Recently, the development of mass spectrometry (MS) techniques, with constant increases in sensitivity and speed of analysis, has facilitated their identification and functional characterization [14, 30–34]. In order to utilize these technologies, histones are first biochemically enriched taking advantage of their highly basic nature. Then, they are proteolyzed with proteases to form short peptides, which are then analysed by MS/MS. The acquired MS/MS spectra are interpreted and converted into amino acid sequences, from which the identity of the original histone protein and the possible presence of post-translational modifications on specific residues can be determined [35]. However, these analyses still remain restricted for a number of reasons. One of these is that the interpretation of MS/MS spectra relies on matching experimental data to theoretical peptide sequences obtained by an *in silico* proteolysis of a list of proteins. Therefore, the content of the theoretical protein sequence database conditions the interpretation of the experimental spectra and the subsequent identification of histones. Classical databases such as Swiss-Prot, trEMBL and NCBI are usually used with success. However, histones have not been precisely annotated in these resources. Manually curated databases such as Swiss-Prot lack several histones, while others, such as trEMBL or NCBI protein database, are more extensively populated with non-reviewed data. The latter contain more histone entries, but the degree of redundancy and the precision of the descriptions can make protein identification results difficult to interpret. Finally, naming of histones has been recently revisited with a new unified nomenclature [36]. The recent release of HistoneDB 2.0 consolidated the sequence information of a large variety of histones and their sequence variants in many organisms [37]. However, it has not yet been integrated in the above databases and the same variant can go by different names. For instance, the coding gene *H2afb1* refers to proteins H2A.L.2 or H2A.Lap3 in the literature and to H2A-Bbd type 1 in the NCBI RefSeq and UniProtKB databases [38, 39]. In addition, a different protein coded by *Gm14920* is also named H2A.Bbd.1 in other publications [40]. Here, a unified name is presented to identify uniquely each ambiguous entry and is also associated with its other names to facilitate its relationship with previously published work.

We have collected redundant histone entries from a number of public databases, gathering >700 entries for mouse and >1000 entries for human histones. We manually curated these lists to obtain a final count of 83 and 85 histone entries for mouse or human, respectively. Their annotations have been revisited to match the current histone nomenclature in accordance with the new resource “HistoneDB 2.0—with variants” [37]. About 30% of these entries have a fuzzy UniProtKB protein annotation, such as “predicted” or “inferred by homology”, and we performed MS analysis to clearly identify several of these imprecisely characterized entries (some of which had formerly been described to be detected by western blot).

## Results

### MS_HistoneDB, a resource containing unique and non-redundant histones

Our initial aim with this work was to generate an exhaustive and non-redundant resource that would facilitate histone analysis by MS. We identified and collected all the information available on human and mouse histones from the public databases of NCBI, Ensembl and UniProtKB (Table 1). This work was aided by the recent release of an updated version of the Histone Database, named “HistoneDB 2.0—with variants” [37]. This database contains 38,664 entries from 1624 species, with 761 and 1039 entries for mouse and human, respectively. In addition, several histones were also considered based on published articles [41–45].

**Table 1:** Histone entries in various publicly accessible databases

	Mouse			Human		
	NCBI HistoneDB 2.0	UniProtKB	This study	NCBI HistoneDB 2.0	UniProtKB	This study
H1	170	26	16	126	14	12
H2A	238	44	37	313	44	35
H2B	121	19	16	239	32	21
H3	151	17	13	189	22	16
H4	81	4	1	172	4	1
Total	761	110	83	1039	1126	85

**Fig. 1:**
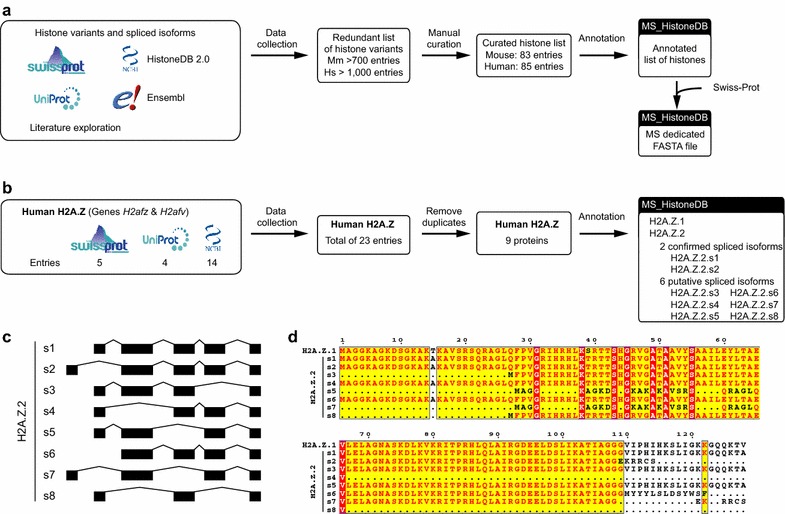
Methodology used to create a manually curated MS_HistoneDB resource. **a** Representation of the dataflow used to generate the MS_HistoneDB resource. **b** Example of the dataflow for human H2A.Z histone variants. The number of entries at each step is indicated. **c** Graphical representation of the exons of human H2A.Z.2 spliced isoforms. **d** Sequence alignment of human H2A.Z variant isoforms

**Fig. 2:**
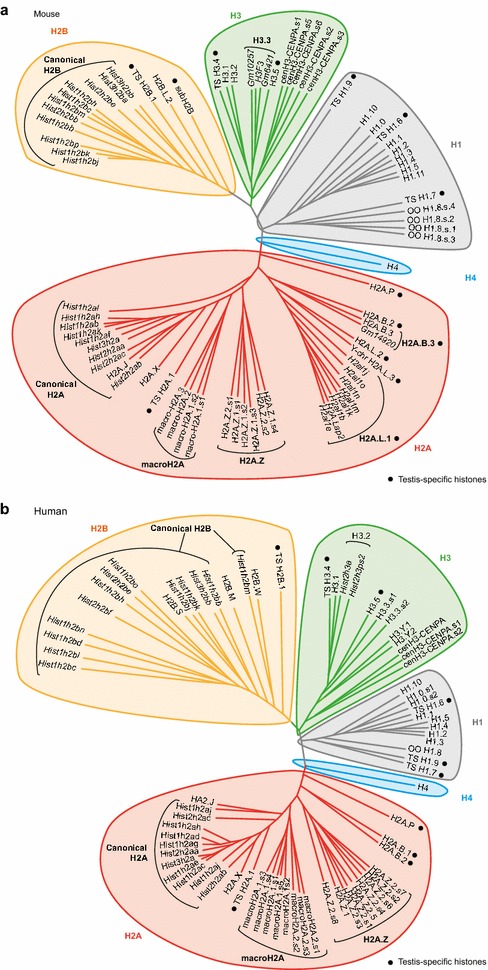
Phylogenetic trees for mouse and human histone entries in MS_HistoneDB. Please note that for clarity, some putative spliced isoforms of canonical histones were not included, as well as other very short spliced isoforms for some histone variants. The full lists are presented in Tables 2 and 3. Gene names are indicated in *italic* to identify histone isoforms grouped under a generic term (see Tables 2 and 3; Additional files 4, 5). *Black dots* highlight testis-specific variants

The dataflow is presented in Fig. 1a. This curating process is exemplified with human H2A.Z on Fig. 1b. A total of 23 entries were collected from Swiss-Prot, Uniprot-trEMBL and NCBI databases. Fourteen were duplicated and removed to obtain nine unique entries, which were annotated as H2A.Z.1 and eight spliced isoforms of H2A.Z.2 using the release R86 of the Ensembl database (Fig. 1c, d). In summary, the following rules were applied. First, each entry is protein-centric and therefore defined by the final product, a unique mature protein. Second, it must be associated with gene, transcript and protein accession numbers in NCBI and/or Ensembl, unless published data document its existence. Third, histone names are not always consistent within the existing public databases. Some were renamed following the Talbert et al. nomenclature and in agreement with the HistoneDB 2.0 resource as detailed in the following sections [36, 37]. The final list of histone entries is presented as a phylogenetic tree in Fig. 2 and in Tables 2 and 3 for mouse and human species, respectively. We did not provide here an extensive review of the functional roles of each histone variant, which are already available elsewhere [4, 21, 23, 36, 46].

**Table 2:** Manually curated list of mouse histones

Histone	Protein name	Entry name for MS analysis	Gene name	UniProtKB	References
H1	H1.1	H1.1	*Hist1h1a*	P43275	[41, 91]
	H1.2	H1.2	*Hist1h1c*	P15864	[41, 92]
	H1.3	H1.3	*Hist1h1d*	P43277	[41]
	H1.4	H1.4	*Hist1h1e*	P43274	[41]
	H1.5	H1.5	*Hist1h1b*	P43276	[41]
	H1.0 (H1°)	H1.0 (H1°)	*H1f0*	P10922	[93]
	TS H1.6 (H1T)	TS H1.6 (H1T)	*Hist1h1t*	Q07133	[54]
	TS H1.7 (H1T2, HANP1)	TS H1.7 (H1T2, HANP1)	*H1fnt*	Q8CJI4	[55, 56]
	OO H1.8 (H1oo)	OO H1.8.s1 (H1oo)	*H1foo*	Q8VIK3	[59]
		OO H1.8.s2	*H1foo*	Q8VIK3-2	[60]
		OO H1.8.s3 (putative spliced isoform)	*H1foo*	E0CZ52	*
		OO H1.8.s4 (putative spliced isoform)	*H1foo*	E0CYL2	Short**
		OO H1.8.s5 (putative spliced isoform)	*H1foo*	A0A0N4SV54	Short*
	TS H1.9 (HILS1)	TS H1.9 (HILS1)	*Hils1*	Q9QYL0	[57, 58]
	H1.10	H1.10	*H1fx*	Q80ZM5	*
	H1.11	H1.11 gene: Gm6970	*Gm6970*	F7DCP6	*
H2A	Canonical H2A	Canonical H2A genes: Hist1h2ab, Hist1h2ac, Hist1h2ad, Hist1h2ae, Hist1h2ag, Hist1h2ai, Hist1h2an, Hist1h2ao, Hist1h2ap	*Hist1h2ab*	P22752	[41]
			*Hist1h2ac*	P22752	[41]
			*Hist1h2ad*	P22752	[41]
			*Hist1h2ae*	P22752	[41]
			*Hist1h2ag*	P22752	[41]
			*Hist1h2ai*	P22752	[41]
			*Hist1h2an*	P22752	[41]
			*Hist1h2ao*	P22752	[41]
			*Hist1h2ap*	P22752	[41]
		Canonical H2A gene: Hist1h2af	*Hist1h2af*	Q8CGP5	[41]
		Canonical H2A gene: Hist1h2ah	*Hist1h2ah*	Q8CGP6	[41]
		Canonical H2A gene: Hist1h2ak	*Hist1h2ak*	Q8CGP7	[41]
		Canonical H2A gene: Hist1h2al	*Hist1h2al*	F8WIX8	*
		Canonical H2A genes: Hist2h2aa1, Hist2h2aa2	*Hist2h2aa1*	Q6GSS7	[41]
			*Hist2h2aa2*	Q6GSS7	[41]
		Canonical H2A gene: Hist2h2ab	*Hist2h2ab*	Q64522	[41]
		Canonical H2A gene: Hist2h2ac	*Hist2h2ac*	Q64523	[41]
		Canonical H2A gene: Hist3h2a	*Hist3h2a*	Q8BFU2	[41]
	H2A.J (putative variant)	H2A.J.s1 (putative variant)	*H2afj*	Q8R1M2	*
		H2A.J.s2 (putative variant, putative spliced isoform)	*H2afj*	A0A0N4SV66	*
	H2A.X	H2A.X	*H2afx*	P27661	[61, 62, 94]
	H2A.Z.1	H2A.Z.1.s1	*H2afz*	P0C0S6	[43]
		H2A.Z.1.s2 (putative spliced isoform)	*H2afz*	Q3UA95	*
		H2A.Z.1.s3 (putative spliced isoform)	*H2afz*	G3UWL7	Short*
		H2A.Z.1.s4 (putative spliced isoform)	*H2afz*	G3UX40	Short**
	H2A.Z.2	H2A.Z.2.s1	*H2afv*	Q3THW5	[43]
		H2A.Z.2.s2 (putative spliced isoform)	*H2afv*	Q8R029	Short*
	Macro-H2A.1	Macro-H2A.1.s1	*H2afy*	Q9QZQ8	[95]
		Macro-H2A.1.s2	*H2afy*	Q9QZQ8-2	[45]
	Macro-H2A.2	Macro-H2A.2	*H2afy2*	Q8CCK0	[96, 97]
	Macro-H2A.3	Macro-H2A.3 (pseudogene) gene: H2afy3	*H2afy3*	Q9D3V6	***
	TS H2A.1	TS H2A.1 (TH2A)	*Hist1h2aa*	Q8CGP4	[41]
	H2A.L.1 (H2A.Lap2)	H2A.L.1 (H2A.Lap2) genes: H2al1a, GH2al1c,H2al1d, H2al1f,H2al1g, H2al1h,H2al1i	*H2al1a*	Q5M8Q2	[38, 39]
			*H2al1c*	Q5M8Q2	[38, 39]
			*H2al1d*	Q5M8Q2	[38, 39]
			*H2al1f*	Q5M8Q2	[38, 39]
			*H2al1g*	Q5M8Q2	[38, 39]
			*H2al1h*	Q5M8Q2	[38, 39]
			*H2al1i*	Q5M8Q2	[38, 39]
		H2A.L.1 gene: H2al1b	*H2al1b*	A0A087WP11	*
		H2A.L.1 gene: H2al1e	*H2al1e*	Q810S6	*
		H2A.L.1 gene: H2al1j	*H2al1j*	A2BFR3	*
		H2A.L.1 gene: H2al1k	*H2al1k*	J3QP08	*
		H2A.L.1 gene: H2al1m	*H2al1m*	Q9DAD9	*
		H2A.L.1 gene: H2al1n	*H2al1n*	Q497L1	*
		H2A.L.1 gene: H2al1o	*H2al1o*	L7MU04	*
	H2A.L.2 (H2A.Lap3, H2A.B.1)	H2A.L.2 (H2A.Lap3, H2A.B.1) gene: H2afb1	*H2afb1*	Q9CQ70	[38, 39]
	Y-chr H2A.L.3	Y-chr H2A.L.3 genes: H2al2b, H2al2c	*H2al2b*	A9Z055	[98]
			*H2al2c*	A9Z055	[98]
	H2A.P (H2A.L3, H2A.Lap4)	H2A.P (H2A.L3, H2A.Lap4) gene: Hypm	*Hypm*	Q9CR04	[38, 39]
	H2A.B.2	H2A.B.2 gene: H2afb2	*H2afb2*	S4R1M3	[40, 99]
	H2A.B.3	H2A.B.3 gene: H2afb3	*H2afb3*	S4R1G7	[40, 99]
		H2A.B.3 (H2A.Lap1) gene: Gm14920	*Gm14920*	S4R1E0	[39, 40, 99]
H2B	Canonical H2B	Canonical H2B gene: Hist1h2bb	*Hist1h2bb*	Q64475	[41]
		Canonical H2B genes: Hist1h2bc, Hist1h2be, Hist1h2bg	*Hist1h2bc*	Q6ZWY9	[41]
			*Hist1h2be*	Q6ZWY9	[41]
			*Hist1h2bg*	Q6ZWY9	[41]
		Canonical H2B genes: Hist1h2bf, Hist1h2bj, Hist1h2bl, Hist1h2bn, Hist1h2bq, Hist1h2br	*Hist1h2bf*	P10853	[41]
			*Hist1h2bj*	P10853	[41]
			*Hist1h2bl*	P10853	[41]
			*Hist1h2bn*	P10853	[41]
			*Hist1h2bq*	P10853	[41]
			*Hist1h2br*	P10853	[41]
		Canonical H2B genes: Hist1h2bq, Hist1h2br (putative spliced isoform)	*Hist1h2bq*	Q8CBB6	*
			*Hist1h2br*	Q8CBB6	*
		Canonical H2B gene: Hist1h2bh	*Hist1h2bh*	Q64478	[41]
		Canonical H2B gene: Hist1h2bk	*Hist1h2bk*	Q8CGP1	[41]
		Canonical H2B gene: Hist1h2bm	*Hist1h2bm*	P10854	[41]
		Canonical H2B gene: Hist1h2bp Spliced isoform 1 (main)	*Hist1h2bp*	Q8CGP2	[41]
		Canonical H2B gene: hist1h2bp (putative spliced isoform)	*Hist1h2bp*	Q8CGP2-2	[41]
		Canonical H2B gene: Hist2h2bb	*Hist2h2bb*	Q64525	[41]
		Canonical H2B gene: Hist2h2be	*Hist2h2be*	Q64524	[41]
		Canonical H2B gene: Hist3h2ba	*Hist3h2ba*	Q9D2U9	[41]
		Canonical H2B gene: Hist3h2bb	*Hist3h2bb*	Q8CGP0	*
	TS H2B.1 (TH2B)	TS H2B.1 (TH2B)	*Hist1h2ba*	P70696	[41, 79]
	subH2B (H2BL.1)	subH2B (H2BL.1)	*1700024p04rik*	Q9D9Z7	[38, 100]
	H2B.L.2	H2B.L.2	*H2bfm*	Q9DAB5	[38]
H3	Canonical H3.1	Canonical H3.1	*Hist1h3a*	P68433	[41]
			*Hist1h3g*	P68433	[41]
			*Hist1h3h*	P68433	[41]
			*Hist1h3i*	P68433	[41]
	Canonical H3.2	Canonical H3.2	*Hist1h3b*	P84228	[41]
			*Hist1h3c*	P84228	[41]
			*Hist1h3d*	P84228	[41]
			*Hist1h3e*	P84228	[41]
			*Hist1h3f*	P84228	[41]
			*Hist2h3b*	P84228	[41]
			*Hist2h3c1*	P84228	[41]
			*Hist2h3c2*	P84228	[41]
	H3.3	H3.3 genes: H3f3a, H3f3b	*H3f3a*	P84244	[101, 102]
			*H3f3b*	P84244	[101, 102]
		H3.3 gene: Gm6421	*Gm6421*	EDL18362.1	[103]
		H3.3 gene: Gm10257	*Gm10257*	XP_003084990.1	[103]
	cenH3-CENPA	cenH3-CENPA.s1	*Cenpa*	O35216	[104]
		cenH3-CENPA.s2 (putative spliced isoform)	*Cenpa*	D6RCV6	Short**
		cenH3-CENPA.s3 (putative spliced isoform)	*Cenpa*	D6RJ71	**
		cenH3-CENPA.s4 (putative spliced isoform)	*Cenpa*	A0A0G2JEV0	*
		cenH3-CENPA.s5 (putative spliced isoform)	*Cenpa*	A0A0G2JGI2	*
		cenH3-CENPA.s6 (putative spliced isoform)	*Cenpa*	A0A0G2JEV2	**
	H3.5	H3.5	*H3f3c*	P02301	***
	TS H3.4 (H3T)	TS H3.4 (H3T)	*Gm12260*	NP_001304932.1	[74]
H4	H4	H4	*Hist1h4a*	P62806	[41, 105]
			*Hist1h4b*	P62806	[41, 105]
			*Hist1h4c*	P62806	[41, 105]
			*Hist1h4d*	P62806	[41, 105]
			*Hist1h4f*	P62806	[41, 105]
			*Hist1h4h*	P62806	[41, 105]
			*Hist1h4i*	P62806	[41, 105]
			*Hist1h4j*	P62806	[41, 105]
			*Hist1h4k*	P62806	[41, 105]
			*Hist1h4m*	P62806	[41, 105]
			*Hist1h4n*	P62806	[41, 105]
			*Hist2h4*	P62806	[41, 105]
			*Hist4h4*	P62806	[41, 105]

Their protein names have been adapted to improve their identification and analysis by mass spectrometry. Indeed, the column “Entry name for MS analysis” represents the information present in the FASTA file (Additional file 1) used as a database to identify peptides and proteins after an MS analysis. The last column indicates studies on histones that described evidence of transcript and/or protein existence. For the sake of completeness, histone entries lacking a related publication were retained and the classification currently proposed by the Ensembl database was specified, as follows

* “Protein coding”, genes and/or transcript that contains an open reading frame (ORF)

** “Nonsense mediated decay”, transcript is thought to undergo nonsense mediated decay

*** “Pseudogene”, genes containing frameshift and/or stop codon(s) that disrupt the ORF

The term “Short” indicates that the putative protein is significantly smaller than conventional histones; its incorporation into chromatin and its biological function is then doubtful. Additional file 4 presents links to gene, transcripts and protein entries to Ensembl and UniProtKB databases

**Table 3:** Manually curated list of human histones

Histone	Protein name	Entry name for MS analysis	Gene name	UniProtKB Accession	References
H1	H1.1	H1.1	*Hist1h1a*	Q02539	[41, 106, 107]
	H1.2	H1.2	*Hist1h1c*	P16403	[41, 106, 107]
	H1.3	H1.3	*Hist1h1d*	P16402	[41, 106, 107]
	H1.4	H1.4	*Hist1h1e*	P10412	[41]
	H1.5	H1.5	*Hist1h1b*	P16401	[41, 108]
	H1.0 (H1°)	H1.0 (H1°)	*H1f0*	P07305	[109]
	TS H1.6 (H1t)	TS H1.6 (H1t)	*Hist1h1t*	P22492	[41]
	TS H1.7 (H1T2, HANP1)	TS H1.7 (H1T2, HANP1)	*H1fnt*	Q75WM6	[110]
	OO H1.8 (H1oo)	OO H1.8.s1 (H1oo)	*H1foo*	Q8IZA3-1	[111, 112]
		OO H1.8.s2 (putative spliced isoform)	*H1foo*	Q8IZA3-2	*
	TS H1.9 (Hils)	TS H1.9 (Hils)	*Hils1*	P60008	[57]
	H1.10	H1.10	*H1fx*	Q92522	[113]
H2A	Canonical H2A	Canonical H2A genes: Hist1h2ag, Hist1h2ai, Hist1h2ak, Hist1h2al, Hist1h2am	*Hist1h2ag*	P0C0S8	[41]
			*Hist1h2ai*	P0C0S8	[41]
			*Hist1h2ak*	P0C0S8	[41]
			*Hist1h2al*	P0C0S8	[41]
			*Hist1h2am*	P0C0S8	[41]
		Canonical H2A gene: Hist1h2ac	*Hist1h2ac*	Q93077	[41]
		Canonical H2A gene: Hist1h2ad	*Hist1h2ad*	P20671	[41]
		Canonical H2A gene: Hist1h2ae	*Hist1h2ae*	P04908	[41]
		Canonical H2A gene: Hist1h2ah	*Hist1h2ah*	Q96KK5	[41]
		Canonical H2A gene: Hist1h2aj	*Hist1h2aj*	Q99878	[41]
		Canonical H2A gene:Hist2h2aa4	*Hist1h2aa4*	Q6FI13	[41]
		Canonical H2A gene: Hist2h2ab	*Hist2h2ab*	Q8IUE6	[41]
		Canonical H2A gene: Hist2h2ac	*Hist2h2ac*	Q16777	[41]
		Canonical H2A gene: Hist3h2a	*Hist3h2a*	Q7L7L0	[41]
		Canonical H2A (pseudogene)	*Hist1h2Aps4*	Q92646	***
	H2A.J (putative variant)	H2A.J.s1	*H2afj*	Q9BTM1-1	**
		H2A.J.s2 (putative spliced isoform)	*H2afj*	Q9BTM1-2	**
		H2A.J.s3 (putative spliced isoform)	*H2afj*	H0YFX9	Short**
	H2A.X	H2A.X	*H2afx*	P16104	[61, 114]
	H2A.Z.1	H2A.Z.1	*H2afz*	P0C0S5	[115, 116]
	H2A.Z.2	H2A.Z.2.s1	*H2afv*	Q71UI9-1	[116]
		H2A.Z.2.s2	*H2afv*	Q71UI9-2	[65]
		H2A.Z.2.s3 (putative spliced isoform)	*H2afv*	Q71UI9-4	[65]
		H2A.Z.2.s4 (putative spliced isoform)	*H2afv*	Q71UI9-5	[65]
		H2A.Z.2.s5 (putative spliced isoform)	*H2afv*	Q71UI9-3	[65]
		H2A.Z.2.s6 (putative spliced isoform)	*H2afv*	C9J0D1	*
		H2A.Z.2.s7 (putative spliced isoform)	*H2afv*	C9J386	Short*
		H2A.Z.2.s8 (putative spliced isoform)	*H2afv*	E5RJU1	Short*
	macroH2A.1	macroH2A.1.s1	*H2afy*	O75367	[117]
		macroH2A.1.s2	*H2afy*	O75367-2	[66]
		macroH2A.1.s3 (putative spliced isoform)	*H2afy*	B4DJC3	*
		macroH2A.1.s4 (putative spliced isoform)	*H2afy*	D6RCF2	***
		macroH2A.1.s5 (putative spliced isoform)	*H2afy*	O75367-3	*
	macroH2A.2	macroH2A.2.s1	*H2afy2*	Q9P0M6	[96, 97]
		macroH2A.2.s2 (putative spliced isoform)	*H2afy2*	Q5SQT3	*
	TS H2A.1 (TH2A)	TS H2A.1 (TH2A)	*Hist1h2aa*	Q96QV6	[71]
	H2A.B.1	H2A.B.1	*H2afb1*	P0C5Y9	[118, 119]
	H2A.B.2	H2A.B.2	*H2afb2*	P0C5Z0	*
			*H2afb3*		
	H2A.P	H2A.P	*Hypm*	O75409	*
H2B	Canonical H2B	Canonical H2B gene: Hist1h2bb	*Hist1h2bb*	P33778	[41]
		Canonical H2B genes: Hist1h2bc, Hist1h2be, Hist1h2bf, Hist1h2bg, Hist1h2bi	*Hist1h2bc*	P62807	[41]
			*Hist1h2be*	P62807	[41]
			*Hist1h2bf*	P62807	[41]
			*Hist1h2bg*	P62807	[41]
			*Hist1h2bi*	P62807	[41]
		Canonical H2B gene: Hist1h2bd	*Hist1h2bd*	P58876	[41]
		Canonical H2B gene: Hist1h2bh	*Hist1h2bh*	Q93079	[41]
		Canonical H2B gene: Hist1h2bj	*Hist1h2bj*	P06899	[41]
		Canonical H2B gene: Hist1h2bj (putative spliced isoform)	*Hist1h2bj*	U3KPT8	*
		Canonical H2B gene: Hist1h2bk	*Hist1h2bk*	O60814	[41]
		Canonical H2B gene: Hist1h2bl	*Hist1h2bl*	Q99880	[41]
		Canonical H2B gene: Hist1h2bm	*Hist1h2bm*	Q99879	[41]
		Canonical H2B gene: Hist1h2bn	*Hist1h2bn*	Q99877	[41]
		Canonical H2B gene: Hist1h2bn (putative spliced isoform)	*Hist1h2bn*	U3KQK0	[41]
		Canonical H2B gene: Hist1h2bo	*Hist1h2bo*	P23527	[41]
		Canonical H2B gene: Hist2h2be	*Hist2h2be*	Q16778	[41]
		Canonical H2B gene: Hist2h2bf (putative spliced isoform)	*Hist2h2bf*	Q5QNW6	*
		Canonical H2B gene: Hist2h2bf (putative spliced isoform)	*Hist2h2bf*	Q5QNW6-2	*
		Canonical H2B gene: Hist3h2bb	*Hist3h2bb*	Q8N257	[41]
	H2B.S (putative variant)	H2B.S (putative variant)	*H2bfs*	P57053	*
	H2B.M (putative variant)	H2B.M.s1 (putative variant)	*H2bfm*	P0C1H6	*
		H2B.M.s2 (putative variant, putative spliced isoform)	*H2bfm*	A9UJN3	Short*
	H2B.W	H2B.W	*H2bfwt*	Q7Z2G1	[72, 120, 121]
	TS H2B.1 (TH2B)	TS H2B.1 (TH2B)	*Hist1h2ba*	Q96A08	[41, 71]
H3	Canonical H3.1	Canonical H3.1 genes: Hist1h3a, Hist1h3b, Hist1h3c, Hist1h3d, Hist1h3e, Hist1h3f, Hist1h3g, Hist1h3h, Hist1h3i, Hist1h3j	*Hist1h3a*	P68431	[41]
			*Hist1h3b*	P68431	[41]
			*Hist1h3c*	P68431	[41]
			*Hist1h3d*	P68431	[41]
			*Hist1h3e*	P68431	[41]
			*Hist1h3f*	P68431	[41]
			*Hist1h3g*	P68431	[41]
			*Hist1h3h*	P68431	[41]
			*Hist1h3i*	P68431	[41]
			*Hist1h3j*	P68431	[41]
	Canonical H3.2	Canonical H3.2 genes: Hist2h3a, Hist2h3c, Hist2h3d	*Hist2h3a*	Q71DI3	[41]
			*Hist2h3c*	Q71DI3	[41]
			*Hist2h3d*	Q71DI3	[41]
		Canonical H3.2 (pseudogene)	*Hist2h3ps2*	Q5TEC6	*
	H3.3	H3.3.s1	*H3f3a*	P84243	[122, 123]
			*H3f3b*	P84243	[122, 123]
		H3.3.s2 (putative spliced isoform)	*H3f3a*	B4DEB1	*
			*H3f3b*	B4DEB1	*
		H3.3.s3 (putative spliced isoform)	*H3f3b*	K7EK07	*
		H3.3.s4 (putative spliced isoform)	*H3f3b*	K7EMV3	*
		H3.3.s5 (putative spliced isoform)	*H3f3b*	K7EP01	*
		H3.3.s6 (putative spliced isoform)	*H3f3b*	K7ES00	*
	H3.Y.1	H3.Y.1	*H3.Y*	Translated from NG_012784.2	[44]
	H3.Y.2 (H3.X)	H3.Y.2 (H3.X)	*H3.X*	Translated from NG_023411.2	[44]
	H3.5	H3.5	*H3f3c*	Q6NXT2	[75]
	cenH3-CENPA	cenH3 - CENPA	*Cenpa*	P49450-1	[124]
		cenH3.s1 (putative spliced isoform)	*Cenpa*	P49450-2	*
		cenH3.s2 (putative spliced isoform)	*Cenpa*	F8WD88	Short**
	TS H3.4 (H3t)	TS H3.4 (H3t)	*Hist3h3*	Q16695	[41]
H4	H4	H4	*Hist1h4a*	P62805	[41]
			*Hist1h4b*	P62805	[41]
			*Hist1h4c*	P62805	[41]
			*Hist1h4d*	P62805	[41]
			*Hist1h4e*	P62805	[41]
			*Hist1h4f*	P62805	[41]
			*Hist1h4h*	P62805	[41]
			*Hist1h4i*	P62805	[41]
			*Hist1h4j*	P62805	[41]
			*Hist1h4k*	P62805	[41]
			*Hist1h4l*	P62805	[41]
			*Hist2h4a*	P62805	[41]
			*Hist2h4b*	P62805	[41]
			*Hist4h4*	P62805	[41]

Please refer to the Table 2 for legend. The corresponding FASTA file is presented as Additional file 2. Additional file 5 presents links to gene, transcripts and protein entries to Ensembl and UniProtKB databases

### Canonical histones

Canonical histones constitute the bulk of the proteins that organize DNA into chromatin. They are synthesized and incorporated into chromatin during replication [41]. Their expression is carefully regulated to provide enough proteins to be loaded onto newly synthesized DNA while preventing the accumulation of free histones [47, 48]. For this reason, they are denominated “replication-dependent” and their mRNA adopts a unique organization (for review, see [49]). They are the only RNA polymerase II transcripts which are not polyadenylated but instead possess a 3′ stem-loop, formed during the maturation of their mRNA and which is essential for their regulation [49]. However, polyadenylation events of replication-dependent histone mRNA have recently been identified in terminally differentiated cells and suggested to provide a replacement pool of canonical histones [50].

H2A and H2B canonical histones have minor sequence variations, and it is not clear yet whether these have a functional significance [51]. MS analysis can differentiate between these isoforms and their denomination had to be adapted for proteomic analysis. Here, we propose that canonical H2A and H2B isoforms can be regrouped under the generic term “canonical H2A” or “canonical H2B”, complemented by the gene name of each isoform (Tables 2, 3).

### Histone variants are mostly replication-independent

In contrast to canonical histones, almost all histone variants are synthesized independently of the cell cycle and named “replication-independent” [49]. Their mRNA is polyadenylated and these histones are incorporated into chromatin at any time of the cell cycle. Two exceptions are the testis-specific (TS) histone variants TS H2A and TS H2B, which possess a 3′ stem-loop in their mRNA. For this reason, they have been classified as replication-dependent [49] even if expressed in differentiating germ cells which replicate their DNA only once before meiosis.

### Spliced and putative isoforms

More than 40 spliced isoforms for all mouse and human histones are present in the Ensembl database. However, this information, mainly based on transcriptional data, remains questionable; notably whether the corresponding proteins are expressed and incorporated into chromatin is uncertain. Some spliced isoforms correspond to very short isoforms that lack the globular domain and are probably, if expressed, non-functional (mouse: cenH3-CENPA.s2, cenH3-CENPA.s3, cenH3-CENPA.s4, cenH3-CENPA.s5, OO H1.8.s.4, OO H1.8.s.5, H2A.Z.1.s3, H2A.Z.1.s4, H2A.Z.2.s2; human: H2A.J.s3, H2A.Z.2.s7, H2A.Z.2.s8, canonical H2B.s2, cenH3-CENPA.s2). Even though their expression remains highly uncertain, they have been included in MS_HistoneDB for their identification by MS to be possible. Observing the presence of a shorter non-functional sequence at the expense of the full-length histone would indeed constitute interesting information. Following the same rationale, several putative isoforms or pseudogenes have been included in this resource (Tables 2, 3). Their detection by MS will constitute an indispensable step to confirm the expression of their protein form.

### H1 histones

H1 histones (or linker histones) are different from core histones with respect to their structure, function and evolution. Therefore, it is not possible to single out one of its isoforms as canonical. H1 variants are known to encompass isoforms named H1.0–H1.10. H1.1–H1.5 from histone gene cluster 1 and orphan genes H1.0 (H1°) and H1.10 are usually referred to as somatic variants [36]. The linker variant H1.0 has been described to be involved in cell differentiation (for review [2, 52]). H1.10 has been identified in human and plays an essential role for mitotic progression [53]. H1 variants also include the TS proteins TS H1.6 [54], TS H1.7 [55, 56], TS H1.9 [57, 58] and the oocyte-specific OO H1.8 variant [59, 60]. Finally, a new mouse entry, H1.11, was identified here while performing an *in silico* search using sequence alignments.

### H2A variants

H2A variants comprise H2A.X, H2A.Z, macro-H2A and a number of TS variants, TS H2A, H2A.L/H2A.P and H2A.B.

Only one H2A.X protein has been described; this variant is involved in double-strand break repair, genome stability and chromatin remodelling and silencing in male meiosis [61–64]. H2A.Z is involved in transcription regulation and is encoded by two different genes, *H2afz* and *H2afv* [43]. In mouse, four putative spliced isoforms may be expected in addition to the two original sequences, while in human eight H2A.Z.2 isoforms have been suggested, of which two have been demonstrated to be stable at the protein level [65]. The specific functional roles of these isoforms are not well understood yet, but in some specific tissues, such as in the brain, some H2A.Z spliced isoforms could provide context-specific signalling information [65].

Macro-H2A is the largest histone variant with a long C-terminal domain [66]. This histone variant is associated with transcription repression, although recent evidence suggests that in some conditions it may also promote transcription (reviewed in [51]). Macro-H2A is known to be encoded by two or three different genes, for human and mouse, respectively, some of which are differentially spliced. These variable forms allow differential binding of NAD [67].

Finally, many H2A variants are specifically expressed in the testis. First, TS H2A.1 was originally identified in 1982 in the testis, where it plays an important role and was later detected in the ovary [41, 68–71].

**Fig. 3:**
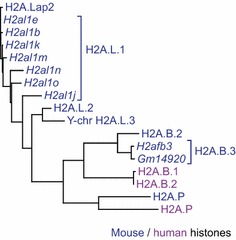
Phylogenetic tree of the mouse and human H2A.L, H2A.B and H2A.P histone variants

Fourteen other mouse TS H2A variants have been grouped into three main classes, H2A.L, H2A.B and H2A.P (Fig. 3). This class also regroups human variants, with two H2A.B and one H2A.P proteins (Fig. 3). They are involved in transcription regulation and the final chromatin reorganization during post-meiotic differentiation of sperm cells [26, 38, 39]. The mouse variants have been described by different research groups [38, 39], and a denomination used here follows previous publications [36, 37]. When potential protein products of different genes have only minor sequence variations and no functional difference has been characterized, we grouped them under the name with the same number suffix (e.g. H2A.L.1); however, the gene name is provided in the name of the entry as the second qualifier. Future studies might warrant splitting of such groups of proteins if functional differences between the members are detected. Currently, H2A.B.2 and H2A.B.3 are proposed to be numbered following their gene name, i.e. *H2AFB2* and *H2AFB3*, respectively. In 2007, new TS H2A variants were identified and named H2AL1 and H2AL2. [38]. A few years later, these histones were independently identified and named H2A.Lap2 and H2A.Lap3, respectively [39]. This latter work also reported the identification and functional characterization of a third member baptized H2A.Lap1 which falls into H2A.B group and is proposed to be regrouped with the highly similar protein H2A.B.3. H2A.L.3 was originally identified by S. Khochbin’s group [38] and is the same as H2A.Lap4, also identified by D. Tremethick’s group [39]. However, it forms a separate phylogenetic clade in placental mammals and is named H2A.P here according to [36].

### H2B variants

Variants TS H2B.1, H2B.L and H2B.W were first identified as TS. In the testes, these proteins are involved in the chromatin-to-protamine transition [38, 69, 72, 73]. Then, TS H2B.1 and TS H2A.1 were also identified in human oocytes, where they favour the generation of induced pluripotent cells [70, 71]. In human, some genes (e.g. *H2BFM*, *H2BFS*) still await characterization and have been denoted as putative variants in this work (Table 3).

### H3 variants

H3 has several isoforms: H3.1 and H3.2 are replication-dependent; H3.3 is considered to be a replication-independent histone variant, while TS H3.4 and H3.5 are TS [74, 75]. Several new isoforms of H3.3 were included in the database developed here along with two other human H3 histone variants, H3.X and H3.Y [44].

CenH3/CENPA is a well-known centromeric H3 variant with many spliced isoforms. Its name has been the subject of heated discussion, which is out of the scope of our work [36, 76, 77]. We therefore propose to use both names, cenH3-CENPA, until a consensus has been reached by the community.

### Generation of MS-based databases

De novo MS data interpretation methods are naive and do not rely on pre-existing databases. However, MS data acquired on histones are generally matched to a database containing all the protein sequences that could theoretically be found in the sample. Using this approach, a given histone protein cannot be identified if its sequence is not present in the database explored by the MS/MS data interpretation software. We used MS_HistoneDB to create a new search space dedicated to the analysis of histones. Basically, mouse or human non-redundant and well-annotated Swiss-Prot FASTA files were cleared of their histone sequences and then repopulated using MS_HistoneDB. This resource is included as Additional files 1 and 2, providing resources to study histones in mouse and human samples, respectively.

### Identification of new histones in mouse

About 30% of the proteins in MS_HistoneDB have imprecise protein annotations in UniProtKB and are presented in Tables 4 and 5. These tables regroup histones that are annotated in the UniProtKB and NCBI databases as “inferred from homology” or “predicted”. Even though a certain number of these histones have already been described in publications, which provide clear evidence of their existence at mRNA and protein levels, they may not have been identified by MS yet. This could explain their poor annotation status in UniProtKB.

**Table 4:** Mouse histone variants with poor annotation status in the UniProtKB database

Names	Accession number	Protein status	Method of detection			References
			This study (number of identified peptides)	Other MS-based studies	Not MS-based studies	
H1.0 (H1°)	P10922	Transcript	Yes (5)	Yes	RT-PCR; WB	[128]
TS H1.7 (H1T2, HANP1)	Q8CJI4	Transcript	Yes (6)		NB; WB	[55]
OO H1.8 (H1oo)	Q8VIK3	Transcript			WB	[59, 60, 129]
H1.11	F7DCP6	Inferred				
Macro-H2A.3	Q9D3V6	Transcript				
H2A.L.1 gene: *H2al1b*	A0A087WP11	Inferred	Yes (1)			
H2A.L.1 gene: *H2al1j*	A2BFR3	Inferred				
H2A.L.1 gene: *H2al1* *k*	J3QP08	Inferred				
H2A.L.1 gene: *H2al1* *m*	Q9DAD9	Transcript				
H2A.L.1 gene: *H2al1n*	Q497L1	Transcript	Yes (2)			
H2A.L.1 gene: *H2al1o*	L7MU04	Inferred				
H2A.L.2 (H2A.B1,H2A.Lap3)	Q9CQ70	Transcript	Yes (3)	Yes	RT-PCR; NB; WB	[38, 39, 125]
Y-chr H2A.L.3	A9Z055	Transcript			RT-PCR	[98]
H2A.P (H2A.L3,H2A.Lap4,)	Q9CR04	Inferred			RT-PCR	[38, 39]
H2A.B.2	S4R1M3	Inferred			RT-PCR; WB	[40, 99]
H2A.B.3 gene: *H2afb3*	S4R1G7	Inferred			RT-PCR; WB	[40, 99]
H2A.B.3 (H2A.Lap1) gene: *GM14920*	S4R1E0	Inferred			RT-PCR; WB	[39, 40, 99]
H2B.L.2	Q9DAB5	Transcript	Yes (4)	Yes	RT-PCR; WB	[38]
H3.3 gene: *GM6421*	EDL18362.1	Predicted	Yes (1)	Yes	RT-PCR	[103]
H3.3 gene: *GM10257*	XP_003084990.1	Record removed	Yes (1)		RT-PCR	[103]
CENPA-cenH3	O35216	Transcript		Yes	qPCR; WB	[126, 127]
H3.5	P02301	Inferred			RT-PCR	
TS H3.4 (H3t)	NP_001304932.1	Predicted		Yes	RT-PCR	[103]

The “protein status” was retrieved from UniProtKB: “Evidence at transcript level” (noted “Transcript”) or “Inferred from homology” (noted “Inferred”, update of July 2016). Three variants are predicted in NCBI database and are absent in UniProtKB. Information about the detection of some variants at the mRNA level (e.g. by RT-PCR) or at the protein level (e.g. by WB or MS) was further completed with publications and compared to the MS identification results obtained in the present study

*NB* northern blot, *WB* western blot, *MS* mass spectrometry

**Table 5:** Human histone variants with poor annotation status in the UniProtKB database

Name (other names)	Protein status	Accession number	Method of detection	References
TS H1.6 (H1t)	Transcript	P22492	WB	[41]
TS H1.7 (H1T2, HANP1)	Transcript	Q75WM6	NB; WB	[110]
OO H1.8 (H1oo)	Transcript	Q8IZA3-1	RT-PCR	[111, 112]
H2A.1.ps	Inferred	Q92646		
H2A.B.1 (H2A.Bbd)	Transcript	P0C5Y9	WB	[118, 119, 130]
H2A.B.2 (H2A.Bbd)	Transcript	P0C5Z0	WB	[118, 119, 130]

The “protein status” was retrieved from UniProtKB: “Evidence at transcript level” (noted “Transcript”) or “Inferred from homology” (noted “Inferred”, update of July 2016**)**. Information about the detection of some variants at the mRNA level (e.g. by RT-PCR) or at the protein level (e.g. by WB or MS) was further completed with publications

*NB* northern blot, *WB* western blot, *MS* mass spectrometry

**Fig. 4:**
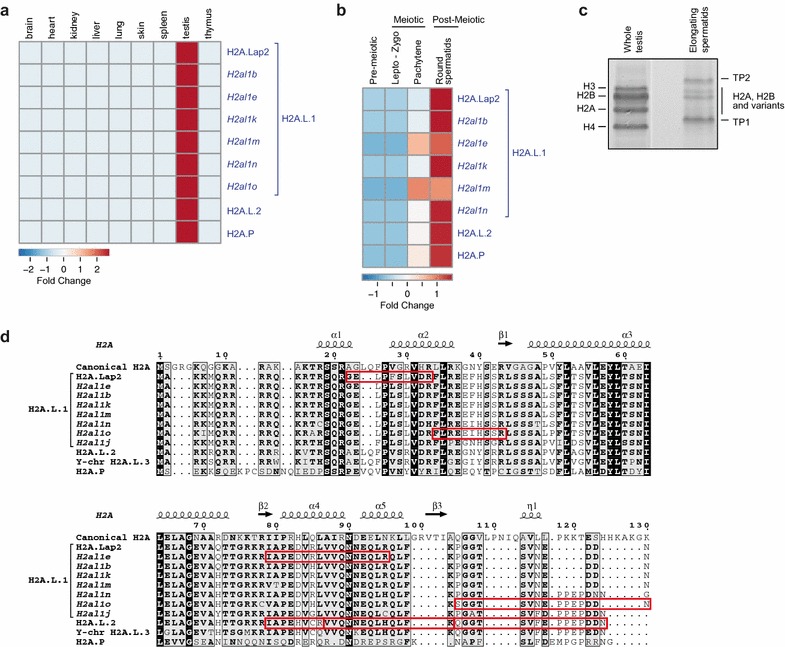
Mouse H2A.L, H2A.B and H2A.P isoforms are expressed in the post-meiotic stages of spermatogenesis. **a** Expression profile of a selection of H2A.L, H2A.B and H2A.P genes across mouse tissues. All of them are testis-specific. Data were extracted using gene names provided in Table 2 and Additional file 4 from a dataset downloaded from Expression Atlas [87] and published in Ref. [82]. **b** Expression profiles of a selection of H2A.L, H2A.B and H2A.P genes during mouse spermatogenesis, revealing a maximum expression in the post-meiotic stage. Data have been obtained from Ref. [83]. *Lepto* leptotene, *Zygo* zygotene. **c** Coomassie-stained SDS-PAGE gel loaded with histones extracted from whole testis and elongating spermatids. **d** Peptides of the mouse H2A.L histone variants identified by mass spectrometry analysis (identified peptides are highlighted in *red boxes*)

At the RNA level, almost all histone variants have been detected in the testis [38–40, 55, 57, 68, 78–81]. Moreover, the expression profile of the mouse H2A.L.1 isoforms which are described in this study has been explored. RNA-seq data from nine mouse tissues have been obtained from a recently published dataset [82]. Expression data were available for 7 out of the 8 H2A.L.1 mouse histone entries and confirm that all of them are mainly expressed in the testis, similarly to H2A.L.2 and H2A.P (Fig. 4a) [38, 39]. Gene expression profiles during spermatogenesis have been obtained from Ref. [83]. It also confirms that H2A.L.1 isoforms are expressed in the post-meiotic stage in spermatids, similarly to H2A.L.2 and H2A.P (Figs. 3c, 4b) [38, 39].

We next decided to test whether MS_HistoneDB would allow the identification by MS of histone entries with imprecise protein annotation using mouse testis. Histones were purified from whole testis or from elongating spermatids (Fig. 4c). Mass spectrometry analysis combined with MS_HistoneDB allowed identification of nine of these poorly annotated proteins (Table 4; Additional file 3). Each newly MS-identified variant was detected by 1–10 specific peptide sequences. The current guidelines for the identification of previously undetected human proteins (“missing proteins”) require the identification of two different peptide sequences of at least nine amino acids in length [84]. To stringently apply the same rules to validate new histone variants would be demanding, given the very high level of sequence homology between some variants. However, out of the nine histone variants detected here for the first time at the protein level by MS-based approaches, six were identified with at least two non-overlapping peptides of length ≥9 amino acids. Almost all the newly identified variants are TS. This analysis thus confirmed the existence of H2A.L.1 encoded by *H2al1b* in mouse testes (Fig. 4d). In addition, this analysis confirmed the existence of the histone variant H3.3 encoded by the gene *Gm10257*, for which a specific peptide has been identified, even if its corresponding NCBI protein record has been recently removed (XP_003084990.1).

Other variants may not be detectable by MS in our analysis. For example, a trypsin digestion does not generate any peptides distinguishing mouse *H2al1k* protein from highly homologous H2A.L.1 variants. Its specific detection would require a more extensive analytical work, e.g. using an alternative protease for protein sample processing, which is beyond the scope of the current work.

## Conclusions

MS is a powerful technique to identify histones, their variants and their post-translational modifications but relies on databases with contradictory naming and excessive redundancy. Here we exhaustively collected histone sequences for mouse and human and used manual curation to establish a protein-centric list, MS_HistoneDB, dedicated to the proteomic study of mouse and human histones. Histone variants whose protein status is uncertain in UniProtKB and NCBI but whose protein existence has been established by experimental evidence described in the literature have been included. This work confirmed the expression of isoforms of previously identified TS histone variants and allowed the detection of one H3.3 isoform whose status was so uncertain that its record had been deleted from the NCBI protein database. We hope that this resource will facilitate the study of histone variants, especially by MS, and their functional roles in physiological and pathological contexts.

## Methods

### Phylogenetic tree representation

Multiple sequence alignments of mouse and human histones were performed using Clustal Omega [85]. Tree data were downloaded in aln format and displayed with iTOLv3 [86].

### Analysis of RNA-seq data

Tissue-specific expression data were obtained from Huntley et al. [82] through the Expression Atlas Repository [87]. RNA-seq data at different stages of spermatogenesis were obtained from da Cruz et al. [83]. Data were imported and treated in R using the pheatmap library (https://CRAN.R-project.org/package=pheatmap).

### Purification of histones from mouse testis

Histones were extracted from two types of biological samples, namely whole testis and elongated spermatids, to maximize the number of histone variants identified at specific maturation stages of male germ cells. Pure fractions of spermatid nuclei were obtained by sonicating mouse testes, as previously described [38]. Histones were isolated from testis cells and spermatids using sulfuric acid [38] or saline extraction [88]. They were then separated by SDS-PAGE, and proteins were visualized by Coomassie staining.

### Sample preparation and analysis by MS

Histones were reduced and alkylated as described previously [89]. Histones were either derivatized with propionic anhydride before and after in-gel trypsin digestion [90], or only submitted to trypsin digestion [89]. The dried extracted peptides were resuspended in 2.5% acetonitrile and 0.05% trifluoroacetic acid and analysed via online nano-LC–MS/MS using an Ultimate 3000 LC system coupled to an LTQ-Orbitrap instrument (CID fragmentation mode) or a Q Exactive Plus instrument (HCD fragmentation mode) (Thermo Fisher Scientific).

### Protein sequence database search and manual verification

MS RAW files produced by LC–MS/MS analysis of proteolyzed histones were processed as follows. All MS/MS spectra were submitted to the Mascot program (version 2.5.1) for searching against the MS_HistoneDB protein sequence database. The parse rules for MS_HistoneDB Fasta files in Mascot are using the accession rule >\([^]*\) and the description rule \(.*\). In addition, the taxonomy and sequence report sources are indicated as “Swiss-Prot FASTA” and “FASTA file”, respectively. No taxonomy was specified when using MS_HistoneDB with Mascot Daemon.

Classical histone modifications were included in the variable modifications: N-terminal protein acetylation; Lys acetylation; and Lys and Arg mono- or di-methylation. For all Mascot searches, the tolerance on mass measurement was set to 5 ppm for peptides and to 0.6 or 0.025 Da for fragment ions when considering LTQ-Orbitrap or Q Exactive acquisitions, respectively. Up to four tryptic missed cleavages were allowed for samples that were not propionylated in vitro, as trypsin does not cleave acetylated lysine, a frequent modification. The enzyme ArgC and up to two missed cleavages were specified for the interpretation of data acquired on propionylated samples. All MS/MS spectra leading to the identification of tryptic peptides specific to newly described variants were carefully manually examined: all major intensity fragment peaks had to be interpreted in terms of y/b ions; a continuous sequence of at least five amino acids had to be read in all cases for validation. Proteomics data are available from ProteomeXchange (PXD005489).

## Additional files

**Additional file 1.** FASTA file containing MS_HistoneDB mouse entries in a mouse Swiss-Prot FASTA file.

**Additional file 2.** FASTA file containing MS_HistoneDB human entries in a human Swiss-Prot FASTA file.

**Additional file 3.** MS/MS spectra assigned to peptides of H2A and H2B variants with an uncertain protein annotation state in UniProtKB or NCBI.

**Additional file 4.** MS_HistoneDB mouse entries with links to gene, transcript and protein identifiers.

**Additional file 5.** MS_HistoneDB human entries with links to gene, transcript and protein identifiers.

## Abbreviations

CID: collision-induced dissociation
FASTA: text-based format for representing protein sequences
HCD: higher-energy collisional dissociation
MS: mass spectrometry
MS/MS: tandem mass spectrometry, used to fragment ions
LC–MS/MS: coupling between liquid chromatography and tandem mass spectrometry
NB: northern blot
PAGE: polyacrylamide gel electrophoresis
TS: testis-specific
WB: western blot

## References

[1] Luger K, Mäder AW, Richmond RK, Sargent DF, Richmond TJ (1997). Crystal structure of the nucleosome core particle at 2.8 Å resolution. Nature.

[2] Izzo A, Schneider R (2016). The role of linker histone H1 modifications in the regulation of gene expression and chromatin dynamics. Biochim Biophys Acta.

[3] Bednar J, Hamiche A, Dimitrov S (2016). H1–nucleosome interactions and their functional implications. Biochim Biophys Acta.

[4] Venkatesh S, Workman JL (2015). Histone exchange, chromatin structure and the regulation of transcription. Nat Rev Mol Cell Biol.

[5] Seeber A, Gasser SM (2016). Chromatin organization and dynamics in double-strand break repair. Curr Opin Genet Dev.

[6] Yong W-S, Hsu F-M, Chen P-Y (2016). Profiling genome-wide DNA methylation. Epigenet Chromatin.

[7] Almouzni G, Cedar H (2016). Maintenance of epigenetic information. Cold Spring Harb Perspect Biol.

[8] Böhmdorfer G, Wierzbicki AT (2015). Control of chromatin structure by long noncoding RNA. Trends Cell Biol.

[9] Hota SK, Bruneau BG (2016). ATP-dependent chromatin remodeling during mammalian development. Development.

[10] Talbert PB, Henikoff S (2010). Histone variants—ancient wrap artists of the epigenome. Nat Rev Mol Cell Biol.

[11] Badeaux AI, Shi Y (2013). Emerging roles for chromatin as a signal integration and storage platform. Nat Rev Mol Cell Biol.

[12] Smith E, Shilatifard A (2010). The chromatin signaling pathway: diverse mechanisms of recruitment of histone-modifying enzymes and varied biological outcomes. Mol Cell.

[13] Huang H, Sabari BR, Garcia BA, Allis CD, Zhao Y (2014). SnapShot: histone modifications. Cell.

[14] Zhao Y, Garcia BA. Comprehensive catalog of currently documented histone modifications. Cold Spring Harb Perspect Biol. 2015;7:a025064. 10.1101/cshperspect.a025064 PMC4563710 26330523

[15] Bannister AJ, Kouzarides T (2011). Regulation of chromatin by histone modifications. Cell Res.

[16] Antonin W, Neumann H (2016). Chromosome condensation and decondensation during mitosis. Curr Opin Cell Biol.

[17] House NCM, Koch MR, Freudenreich CH (2014). Chromatin modifications and DNA repair: beyond double-strand breaks. Front Genet.

[18] Harr JC, Gonzalez-Sandoval A, Gasser SM (2016). Histones and histone modifications in perinuclear chromatin anchoring: from yeast to man. EMBO Rep.

[19] Yen K, Vinayachandran V, Pugh BF (2013). SWR-C and INO80 chromatin remodelers recognize nucleosome-free regions near +1 nucleosomes. Cell.

[20] Krietenstein N, Wal M, Watanabe S, Park B, Peterson CL, Pugh BF (2016). Genomic nucleosome organization reconstituted with pure proteins. Cell.

[21] Weber CM, Henikoff S (2014). Histone variants: dynamic punctuation in transcription. Genes Dev..

[22] Zink L-M, Hake SB (2016). Histone variants: nuclear function and disease. Curr Opin Genet Dev.

[23] Maze I, Noh K-M, Soshnev AA, Allis CD. Every amino acid matters: essential contributions of histone variants to mammalian development and disease. Nat Rev Genet. 2014;15:259–71. 10.1038/nrg3673 PMC4082118 24614311

[24] Govin J, Caron C, Lestrat C, Rousseaux S, Khochbin S (2004). The role of histones in chromatin remodelling during mammalian spermiogenesis. Eur J Biochem.

[25] Rathke C, Baarends WM, Awe S, Renkawitz-Pohl R (2014). Chromatin dynamics during spermiogenesis. Biochim Biophys Acta.

[26] Boussouar F, Rousseaux S, Khochbin S (2008). A new insight into male genome reprogramming by histone variants and histone code. Cell Cycle.

[27] Moosmann A, Campsteijn C, Jansen PW, Nasrallah C, Raasholm M, Stunnenberg HG (2011). Histone variant innovation in a rapidly evolving chordate lineage. BMC Evol.

[28] Siegel TN, Hekstra DR, Kemp LE, Figueiredo LM, Lowell JE, Fenyo D (2009). Four histone variants mark the boundaries of polycistronic transcription units in *Trypanosoma brucei*. Genes Dev.

[29] Bernhard D, Schlegel M (1998). Evolution of histone H4 and H3 genes in different ciliate lineages. J Mol Evol.

[30] Boyne MT, Pesavento JJ, Mizzen CA, Kelleher NL (2006). Precise characterization of human histones in the H2A gene family by top down mass spectrometry. J Proteome Res.

[31] Phanstiel D, Brumbaugh J, Berggren W, Conard K, Feng X, Levenstein M (2008). Mass spectrometry identifies and quantifies 74 unique histone H4 isoforms in differentiating human embryonic stem cells. Proc Natl Acad Sci USA.

[32] Siuti N, Kelleher NL (2007). Decoding protein modifications using to–down mass spectrometry. Nat Methods.

[33] Huang H, Lin S, Garcia BA, Zhao Y (2015). Quantitative proteomic analysis of histone modifications. Chem Rev.

[34] Tan M, Luo H, Lee S, Jin F, Yang JS, Montellier E (2011). Identification of 67 histone marks and histone lysine crotonylation as a new type of histone modification. Cell.

[35] Cox J, Mann M (2011). Quantitative, high-resolution proteomics for data-driven systems biology. Annu Rev Biochem.

[36] Talbert PB, Ahmad K, Almouzni G, Ausió J, Berger F, Bhalla PL (2012). A unified phylogeny-based nomenclature for histone variants. Epigenet Chromatin.

[37] Draizen EJ, Shaytan AK, Mariño-Ramírez L, Talbert PB, Landsman D, Panchenko AR (2016). HistoneDB 2.0: a histone database with variants—an integrated resource to explore histones and their variants. Database.

[38] Govin J, Escoffier E, Rousseaux S, Kuhn L, Ferro M, Thévenon J (2007). Pericentric heterochromatin reprogramming by new histone variants during mouse spermiogenesis. J Cell Biol.

[39] Soboleva TA, Nekrasov M, Pahwa A, Williams R, Huttley GA, Tremethick DJ (2012). A unique H2A histone variant occupies the transcriptional start site of active genes. Nat Struct Mol Biol.

[40] Ishibashi T, Li A, Eirin-Lopez JM, Zhao M, Missiaen K, Abbott DW (2010). H2A.Bbd: an X-chromosome-encoded histone involved in mammalian spermiogenesis. Nucleic Acids Res.

[41] Marzluff WF, Gongidi P, Woods KR, Jin J, Maltais LJ (2002). The human and mouse replication-dependent histone genes. Genomics.

[42] Gautier T, Abbott DW, Molla A, Verdel A, Ausió J, Dimitrov S (2004). Histone variant H2ABbd confers lower stability to the nucleosome. EMBO Rep.

[43] Dryhurst D, Ishibashi T, Rose KL, Eirín-López JM, McDonald D, Silva-Moreno B (2009). Characterization of the histone H2A.Z-1 and H2A.Z-2 isoforms in vertebrates. BMC Biol.

[44] Wiedemann SM, Mildner SN, Bonisch C, Israel L, Maiser A, Matheisl S (2010). Identification and characterization of two novel primate-specific histone H3 variants, H3.X and H3.Y. J Cell Biol.

[45] Bonisch C, Hake SB (2012). Histone H2A variants in nucleosomes and chromatin: more or less stable?. Nucleic Acids Res.

[46] Vardabasso C, Hasson D, Ratnakumar K, Chung C-Y, Duarte LF, Bernstein E (2014). Histone variants: emerging players in cancer biology. Cell Mol Life Sci.

[47] Sittman DB, Graves RA, Marzluff WF (1983). Histone mRNA concentrations are regulated at the level of transcription and mRNA degradation. Proc Natl Acad Sci.

[48] Günesdogan U, Jäckle H, Herzig A (2014). Histone supply regulates S phase timing and cell cycle progression. Elife.

[49] Marzluff WF, Wagner EJ, Duronio RJ (2008). Metabolism and regulation of canonical histone mRNAs: life without a poly(A) tail. Nat Rev Genet.

[50] Lyons SM, Cunningham CH, Welch JD, Groh B, Guo AY, Wei B (2016). A subset of replication-dependent histone mRNAs are expressed as polyadenylated RNAs in terminally differentiated tissues. Nucleic Acids Res.

[51] Shaytan AK, Landsman D, Panchenko AR (2015). Nucleosome adaptability conferred by sequence and structural variations in histone H2A–H2B dimers. Curr Opin Struct Biol.

[52] Khochbin S (2001). Histone H1 diversity: bridging regulatory signals to linker histone function. Gene.

[53] Takata H, Matsunaga S, Morimoto A, Ono-Maniwa R, Uchiyama S, Fukui K (2007). H1.X with different properties from other linker histones is required for mitotic progression. FEBS Lett.

[54] Drabent B, Bode C, Doenecke D (1993). Structure and expression of the mouse testicular H1 histone gene (H1t). Biochim Biophys Acta (BBA) Gene Struct Expr.

[55] Martianov I, Brancorsini S, Catena R, Gansmuller A, Kotaja N, Parvinen M (2005). Polar nuclear localization of H1T2, a histone H1 variant, required for spermatid elongation and DNA condensation during spermiogenesis. Proc Natl Acad Sci USA.

[56] Tanaka H, Iguchi N, Isotani A, Kitamura K, Toyama Y, Matsuoka Y (2005). HANP1/H1T2, a novel histone H1-like protein involved in nuclear formation and sperm fertility. Mol Cell Biol.

[57] Yan W, Ma L, Burns KH, Matzuk MM (2003). HILS1 is a spermatid-specific linker histone H1-like protein implicated in chromatin remodeling during mammalian spermiogenesis. Proc Natl Acad Sci USA.

[58] Iguchi N, Tanaka H, Yomogida K, Nishimune Y (2003). Isolation and characterization of a novel cDNA encoding a DNA-binding protein (Hils1) specifically expressed in testicular haploid germ cells. Int J Androl.

[59] Tanaka M, Hennebold JD, Macfarlane J, Adashi EY (2001). A mammalian oocyte-specific linker histone gene H1oo: homology with the genes for the oocyte-specific cleavage stage histone (cs-H1) of sea urchin and the B4/H1M histone of the frog. Development.

[60] Tanaka M, Kihara M, Hennebold JD, Eppig JJ, Viveiros MM, Emery BR (2005). H1FOO is coupled to the initiation of oocytic growth. Biol Reprod.

[61] Rogakou EP, Pilch DR, Orr AH, Ivanova VS, Bonner WM (1998). DNA double-stranded breaks induce histone H2AX phosphorylation on serine 139. J Biol Chem.

[62] Celeste A, Difilippantonio S, Difilippantonio MJ, Fernandez-Capetillo O, Pilch DR, Sedelnikova OA (2003). H2AX haploinsufficiency modifies genomic stability and tumor susceptibility. Cell.

[63] Bassing CH, Suh H, Ferguson DO, Chua KF, Manis J, Eckersdorff M (2003). Histone H2AX: a dosage-dependent suppressor of oncogenic translocations and tumors. Cell.

[64] Fernandez-Capetillo O, Mahadevaiah SK, Celeste A, Romanienko PJ, Camerini-Otero RD, Bonner WM (2003). H2AX is required for chromatin remodeling and inactivation of sex chromosomes in male mouse meiosis. Dev Cell.

[65] Bonisch C, Schneider K, Punzeler S, Wiedemann SM, Bielmeier C, Bocola M (2012). H2A.Z.2.2 is an alternatively spliced histone H2A.Z variant that causes severe nucleosome destabilization. Nucleic Acids Res.

[66] Gamble MJ, Kraus WL (2014). Multiple facets of the unique histone variant macroH2A: from genomics to cell biology. Cell Cycle.

[67] Han W, Li X, Fu X (2011). The macro domain protein family: structure, functions, and their potential therapeutic implications. Mutat Res.

[68] Trostle-Weige PK, Meistrich ML, Brock WA, Nishioka K, Bremer JW (1982). Isolation and characterization of TH2A, a germ cell-specific variant of histone 2A in rat testis. J Biol Chem.

[69] Shinagawa T, Huynh LM, Takagi T, Tsukamoto D, Tomaru C, Kwak H-G (2015). Disruption of Th2a and Th2b genes causes defects in spermatogenesis. Development.

[70] Shinagawa T, Takagi T, Tsukamoto D, Tomaru C, Huynh LM, Sivaraman P (2014). Histone variants enriched in oocytes enhance reprogramming to induced pluripotent stem cells. Stem Cell.

[71] Huynh LM, Shinagawa T, Ishii S (2016). Two histone variants TH2A and TH2B enhance human induced pluripotent stem cell generation. Stem Cells Dev.

[72] Churikov D, Siino J, Svetlova M, Zhang K, Gineitis A, Morton Bradbury E (2004). Novel human testis-specific histone H2B encoded by the interrupted gene on the X chromosome. Genomics.

[73] Montellier E, Boussouar F, Rousseaux S, Zhang K, Buchou T, Fenaille F (2013). Chromatin-to-nucleoprotamine transition is controlled by the histone H2B variant TH2B. Genes Dev.

[74] Witt O, Albig W, Doenecke D (1996). Testis-specific expression of a novel human H3 histone gene. Exp Cell Res.

[75] Schenk R, Jenke A, Zilbauer M, Wirth S, Postberg J (2011). H3.5 is a novel hominid-specific histone H3 variant that is specifically expressed in the seminiferous tubules of human testes. Chromosoma.

[76] Earnshaw WC, Cleveland DW (2013). CENP-A and the CENP nomenclature: response to Talbert and Henikoff. Trends Genet.

[77] Earnshaw WC, Allshire RC, Black BE, Bloom K, Brinkley BR, Brown W (2013). Esperanto for histones: CENP-A, not CenH3, is the centromeric histone H3 variant. Chromosome Res.

[78] Unni E (1995). Stage-specific distribution of the spermatid-specific histone 2B in the rat testis. Biol Reprod.

[79] Zalensky AO, Siino JS, Gineitis AA, Zalenskaya IA, Tomilin NV, Yau P (2002). Human testis/sperm-specific histone H2B (hTSH2B). Molecular cloning and characterization. J Biol Chem.

[80] Lin Q, Inselman A, Han X, Xu H, Zhang W, Handel MA (2004). Reductions in linker histone levels are tolerated in developing spermatocytes but cause changes in specific gene expression. J Biol Chem.

[81] Greaves IK, Rangasamy D, Devoy M, Marshall Graves JA, Tremethick DJ (2006). The X and Y chromosomes assemble into H2A.Z-containing [corrected] facultative heterochromatin [corrected] following meiosis. Mol Cell Biol.

[82] Huntley MA, Lou M, Goldstein LD, Lawrence M, Dijkgraaf GJP, Kaminker JS (2016). Complex regulation of ADAR-mediated RNA-editing across tissues. BMC Genomics.

[83] da Cruz I, Rodríguez-Casuriaga R, Santiñaque FF, Farías J, Curti G, Capoano CA (2016). Transcriptome analysis of highly purified mouse spermatogenic cell populations: gene expression signatures switch from meiotic-to postmeiotic-related processes at pachytene stage. BMC Genomics.

[84] Carapito C, Lane L, Benama M, Opsomer A, Mouton-Barbosa E, Garrigues L (2015). Computational and mass-spectrometry-based workflow for the discovery and validation of missing human proteins: application to chromosomes 2 and 14. J Proteome Res.

[85] Sievers F, Wilm A, Dineen D, Gibson TJ, Karplus K, Li W (2011). Fast, scalable generation of high-quality protein multiple sequence alignments using Clustal Omega. Mol Syst Biol.

[86] Letunic I, Bork P (2016). Interactive tree of life (iTOL) v3: an online tool for the display and annotation of phylogenetic and other trees. Nucleic Acids Res.

[87] Petryszak R, Keays M, Tang YA, Fonseca NA, Barrera E, Burdett T (2016). Expression Atlas update—an integrated database of gene and protein expression in humans, animals and plants. Nucleic Acids Res.

[88] Shechter D, Dormann HL, Allis CD, Hake SB (2007). Extraction, purification and analysis of histones. Nat Protoc.

[89] Milbradt J, Kraut A, Hutterer C, Sonntag E, Schmeiser C, Ferro M (2014). Proteomic analysis of the multimeric nuclear egress complex of human cytomegalovirus. Mol Cell Proteomics.

[90] Molden RC, Bhanu NV, LeRoy G, Arnaudo AM, Garcia BA (2015). Multi-faceted quantitative proteomics analysis of histone H2B isoforms and their modifications. Epigenet Chromatin.

[91] Dong Y, Sirotkin AM, Yang YS, Brown DT, Sittman DB, Skoultchi AI (1994). Isolation and characterization of two replication-dependent mouse H1 histone genes. Nucleic Acids Res.

[92] Yang YS, Brown DT, Wellman SE, Sittman DB (1987). Isolation and characterization of a mouse fully replication-dependent H1 gene within a genomic cluster of core histone genes. J Biol Chem.

[93] Alonso A, Breuer B, Bouterfa H, Doenecke D (1988). Early increase in histone H1(0) mRNA during differentiation of F9 cells to parietal endoderm. EMBO J.

[94] Porcher C, Grandchamp B (1995). Structure of the mouse H2A.X gene and physical linkage to the UPS locus on chromosome 9: assignment of the human H2A.X gene to 11q23 by sequence analysis. Genomics.

[95] Pehrson FriedV (1992). MacroH2A, a core histone containing a large nonhistone region. Science.

[96] Costanzi C, Pehrson JR (2001). MACROH2A2, a new member of the MACROH2A core histone family. J Biol Chem.

[97] Chadwick BP, Willard HF (2001). Histone H2A variants and the inactive X chromosome: identification of a second macroH2A variant. Hum Mol Genet.

[98] Ferguson L, Ellis PJI, Affara NA (2009). Two novel mouse genes mapped to chromosome Yp are expressed specifically in spermatids. Mamm Genome.

[99] Chen Y, Chen Q, McEachin RC, Cavalcoli JD, Yu X (2014). H2A.B facilitates transcription elongation at methylated CpG loci. Genome Res.

[100] Aul RB, Oko RJ (2001). The major subacrosomal occupant of bull spermatozoa is a novel histone H2B variant associated with the forming acrosome during spermiogenesis. Dev Biol.

[101] Hraba-Renevey S, Kress M (1989). Expression of a mouse replacement histone H3.3 gene with a highly conserved 3′ noncoding region during SV40- and polyoma-induced Go to S-phase transition. Nucleic Acids Res.

[102] Bramlage B, Kosciessa U, Doenecke D (1997). Differential expression of the murine histone genes H3.3A and H3.3B. Differentiation.

[103] Maehara K, Harada A, Sato Y, Matsumoto M, Nakayama KI, Kimura H (2015). Tissue-specific expression of histone H3 variants diversified after species separation. Epigenet Chromatin.

[104] Kalitsis P, MacDonald AC, Newson AJ, Hudson DF, Choo KH (1998). Gene structure and sequence analysis of mouse centromere proteins A and C. Genomics.

[105] Meier VS, Böhni R, Schumperli D (1989). Nucleotide sequence of two mouse histone H4 genes. Nucleic Acids Res.

[106] Ohe Y, Hayashi H, Iwai K (1989). Human spleen histone H1. Isolation and amino acid sequences of three minor variants, H1a, H1c, and H1d. J Biochem.

[107] Eick S, Nicolai M, Mumberg D, Doenecke D (1989). Human H1 histones: conserved and varied sequence elements in two H1 subtype genes. Eur J Cell Biol.

[108] Ohe Y, Hayashi H, Iwai K (1986). Human spleen histone H1. Isolation and amino acid sequence of a main variant, H1b. J Biochem.

[109] Doenecke D, Tonjes R (1986). Differential distribution of lysine and arginine residues in the closely related histones H1 and H5. Analysis of a human H1 gene. J Mol Biol.

[110] Tanaka H, Matsuoka Y, Onishi M, Kitamura K, Miyagawa Y, Nishimura H (2006). Expression profiles and single-nucleotide polymorphism analysis of human HANP1/H1T2 encoding a histone H1-like protein. Int J Androl.

[111] Tanaka Y, Kato S, Tanaka M, Kuji N, Yoshimura Y (2003). Structure and expression of the human oocyte-specific histone H1 gene elucidated by direct RT-nested PCR of a single oocyte. Biochem Biophys Res Commun.

[112] Mizusawa Y, Kuji N, Tanaka Y, Tanaka M, Ikeda E, Komatsu S (2010). Expression of human oocyte-specific linker histone protein and its incorporation into sperm chromatin during fertilization. Fertil Steril.

[113] Happel N, Schulze E, Doenecke D (2005). Characterisation of human histone H1x. Biol Chem.

[114] Mannironi C, Bonner WM, Hatch CL (1989). H2A.X. a histone isoprotein with a conserved C-terminal sequence, is encoded by a novel mRNA with both DNA replication type and polyA 3′ processing signals. Nucleic Acids Res.

[115] Hatch CL, Bonner WM (1988). Sequence of cDNAs for mammalian H2A.Z, an evolutionarily diverged but highly conserved basal histone H2A isoprotein species. Nucleic Acids Res.

[116] Matsuda R, Hori T, Kitamura H, Takeuchi K, Fukagawa T, Harata M (2010). Identification and characterization of the two isoforms of the vertebrate H2A.Z histone variant. Nucleic Acids Res.

[117] Lee Y, Hong M, Kim JW, Hong YM, Choe YK, Chang SY (1998). Isolation of cDNA clones encoding human histone macroH2A1 subtypes. Biochim Biophys Acta.

[118] Tolstorukov MY, Goldman JA, Gilbert C, Ogryzko V, Kingston RE, Park PJ (2012). Histone variant H2A.Bbd is associated with active transcription and mRNA processing in human cells. Mol Cell.

[119] Chadwick BP, Willard HF (2001). A novel chromatin protein, distantly related to histone H2a, is largely excluded from the inactive X chromosome. J Cell Biol.

[120] Boulard M, Gautier T, Mbele GO, Gerson V, Hamiche A, Angelov D (2006). The NH2 tail of the novel histone variant H2BFWT exhibits properties distinct from conventional H2B with respect to the assembly of mitotic chromosomes. Mol Cell Biol.

[121] Ying H-Q, Scott MB, Zhou-cun A (2012). Relationship of SNP of H2BFWT gene to male infertility in a Chinese population with idiopathic spermatogenesis impairment. Biomarkers.

[122] Wells D, Hoffman D, Kedes L (1987). Unusual structure, evolutionary conservation of non-coding sequences and numerous pseudogenes characterize the human H3.3 histone multigene family. Nucleic Acids Res.

[123] Albig W, Bramlage B, Gruber K, Klobeck H-G, Kunz J, Doenecke D (1995). The human replacement histone H3.3B gene (H3F3B). Genomics.

[124] Sullivan KF, Hechenberger M, Masri K (1994). Human CENP-A contains a histone H3 related histone fold domain that is required for targeting to the centromere. J Cell Biol.

[125] Syed S, Boulard M, Shukla M, Gautier T, Travers A, Bednar J (2009). The incorporation of the novel histone variant H2AL2 confers unusual structural and functional properties of the nucleosome. Nucleic Acids Res.

[126] Palmer DK, O’Day K, Margolis RL (1990). The centromere specific histone CENP-A is selectively retained in discrete foci in mammalian sperm nuclei. Chromosoma.

[127] McGregor M, Hariharan N, Joyo A, Margolis RL, Sussman M (2014). CENP-A is essential for cardiac progenitor cell proliferation. Cell Cycle.

[128] Medrzycki M, Zhang Y, Cao K, Fan Y (2012). Expression analysis of mammalian linker-histone subtypes. J Vis Exp.

[129] Furuya M, Tanaka M, Teranishi T, Matsumoto K, Hosoi Y, Saeki K (2007). H1foo is indispensable for meiotic maturation of the mouse oocyte. J Reprod Dev.

[130] Bao Y, Konesky K, Park Y-J, Rosu S, Dyer PN, Rangasamy D (2004). Nucleosomes containing the histone variant H2A.Bbd organize only 118 base pairs of DNA. EMBO J.

[131] Vizcaíno JA, Csordas A, del-Toro N, Dianes JA, Griss J, Lavidas I (2016). 2016 update of the PRIDE database and its related tools. Nucleic Acids Res.

