# Supplementary material for: MS_HistoneDB, a manually curated resource for proteomic analysis of human and mouse histones
Source: Epigenetics Chromatin. 2017 Jan 10;10:2. doi: 10.1186/s13072-016-0109-x (PMC5223428; doi:10.1186/s13072-016-0109-x)
Supplement: Supplementary file 3 — Additional file 3. MS/MS spectra assigned to peptides of H2A and H2B variants with an uncertain protein annotation state in UniProtKB or NCBI. [file 13072_2016_109_MOESM3_ESM.pdf]

SUPPLEMENTARY DATA

Figure S3. MS/MS spectra assigned to peptides of H2A and H2B variants with an uncertain protein annotation state in UniProt/ NCBI.

The tables contain the masses of the fragment ions expected to be produced by fragmentation of the peptide assigned to the MS/MS spectrum. In red bold font are highlighted the masses that were indeed detected in the experimental spectrum. The classically considered fragment ions of (b, y, b0 = b-H2O, y0 = y-H2O, b* = b-NH3 and y* = y–NH3) types were taken into account to interpret the MS/MS spectra produced on the LTQ-Orbitrap instrument and for a first step of interpretation of the Q-Exactive data. For the latter data (HCD fragmentation mode), a (i.e. b – CO) and z (i.e. y-NH3) ions were considered instead of b* and y* ones and low-mass immonium ions were added to confirm the presence of some specific amino acids in the sequence. Finally, internal fragments devoid of the original N- and C-termini of the peptide were considered: they have been described to be significantly produced by HCD fragmentation and can sometimes allow interpreting quite intense experimental fragments (dx.doi.org/10.1021/pr3007045 | J. Proteome Res. 2012, 11, 5479−5491).

H2A.L.1 (H2A.Lap2; Gene: *H2al1a, GH2al1c,H2al1d, H2al1f,H2al1g, H2al1h,H2al1i*): **GELPFSLVDR**

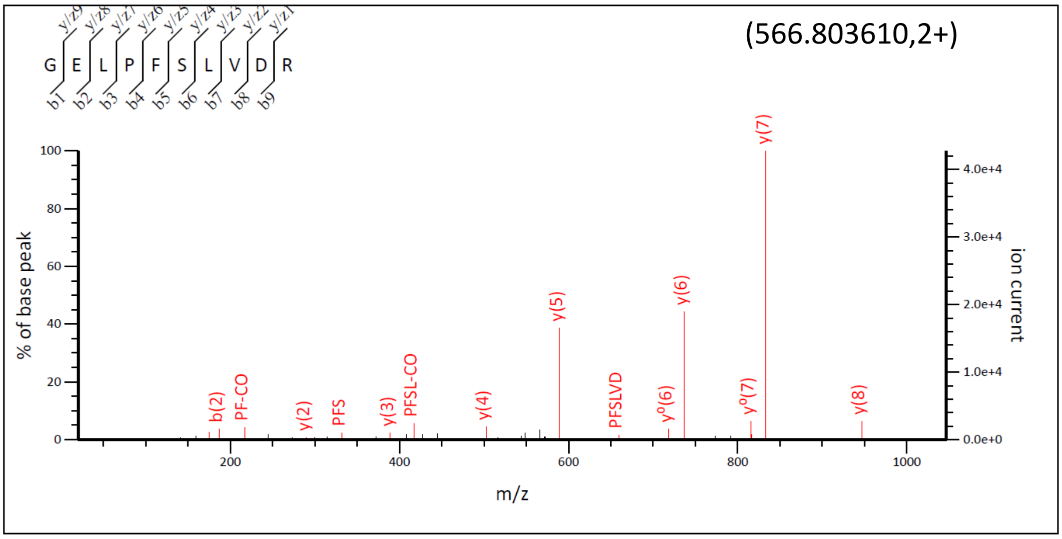

Monoisotopic mass of neutral peptide Mr(calc): 1131.5924
Fixed modifications: Carbamidomethyl (C) (apply to specified residues or termini only)
Ions Score: 76 **Expect:** 3.7e-06
Matches : 21/164 fragment ions using 22 most intense peaks (help)

#	Immon.	a	a^{++}	b	b^{++}	b^{0}	b^{0++}	Seq.	y	y^{++}	y^{0}	y^{0++}	z	z^{++}	#
1	30.0338	30.0338	15.5206	58.0287	29.5180			G							10
2	102.0550	159.0764	80.0418	187.0713	94.0393	169.0608	85.0340	E	1075.5782	538.2928	1057.5677	529.2875	1058.5517	529.7795	9
3	86.0964	272.1605	136.5839	300.1554	150.5813	282.1448	141.5761	L	946.5356	473.7715	928.5251	464.7662	929.5091	465.2582	8
4	70.0651	369.2132	185.1103	397.2082	199.1077	379.1976	190.1024	P	833.4516	417.2294	815.4410	408.2241	816.4250	408.7162	7
5	120.0808	516.2817	258.6445	544.2766	272.6419	526.2660	263.6366	F	736.3988	368.7030	718.3882	359.6978	719.3723	360.1898	6
6	60.0444	603.3137	302.1605	631.3086	316.1579	613.2980	307.1527	S	589.3304	295.1688	571.3198	286.1636	572.3039	286.6556	5
7	86.0964	716.3978	358.7025	744.3927	372.7000	726.3821	363.6947	L	502.2984	251.6528	484.2878	242.6475	485.2718	243.1395	4
8	72.0808	815.4662	408.2367	843.4611	422.2342	825.4505	413.2289	V	389.2143	195.1108	371.2037	186.1055	372.1878	186.5975	3
9	88.0393	930.4931	465.7502	958.4880	479.7476	940.4775	470.7424	D	290.1459	145.5766	272.1353	136.5713	273.1193	137.0633	2
10	129.1135							R	175.1190	88.0631			158.0924	79.5498	1

Seq	ya	yb	Seq	ya	yb	Seq	ya	yb
EL	215.1390	243.1339	ELP	312.1918	340.1867	ELPF	459.2602	487.2551
ELPFS	546.2922	574.2871	ELPFSL	659.3763	687.3712	LP	183.1492	211.1441
LPF	330.2176	358.2125	LPFS	417.2496	445.2445	LPFSL	530.3337	558.3286
LPFSLV	629.4021	657.3970	PF	217.1335	245.1285	PFS	304.1656	332.1605
PFSL	417.2496	445.2445	PFSLV	516.3180	544.3130	PFSLVD	631.3450	659.3399
FS	207.1128	235.1077	FSL	320.1969	348.1918	FSLV	419.2653	447.2602
FSLVD	534.2922	562.2871	SL	173.1285	201.1234	SLV	272.1969	300.1918
SLVD	387.2238	415.2187	LV	185.1648	213.1598	LVD	300.1918	328.1867
VD	187.1077	215.1026						

H2A.L.1 (Gene: *H2alb*):**IAPEDVHLVVQNNEQLR**

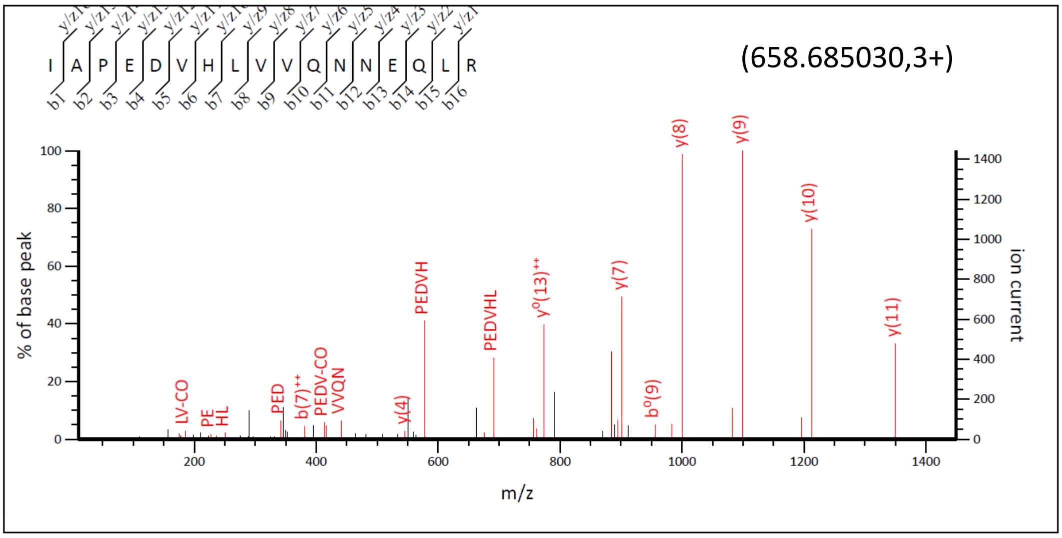

Monoisotopic mass of neutral peptide Mr(calc): 1973.0330
Fixed modifications: Carbamidomethyl (C) (apply to specified residues or termini only)
Ions Score: 65 **Expect:** 3.9e-06
Matches : 33/315 fragment ions using 40 most intense peaks (help)

#	Immon.	a	a^{++}	b	b^{++}	b^0	b^{0++}	Seq.	y	y^{++}	y^0	y^{0++}	z	z^{++}	#
1	86.0964	86.0964	43.5519	114.0913	57.5493			I							17
2	44.0495	157.1335	79.0704	**185.1285**	93.0679			A	1860.9563	930.9818	1842.9457	921.9765	1843.9297	922.4685	16
3	70.0651	254.1863	127.5968	282.1812	141.5942			P	1789.9191	**895.4632**	1771.9086	886.4579	1772.8926	886.9499	15
4	102.0550	383.2289	192.1181	411.2238	206.1155	393.2132	197.1103	E	1692.8664	846.9368	1674.8558	837.9315	1675.8398	838.4236	14
5	88.0393	498.2558	249.6316	526.2508	263.6290	508.2402	254.6237	D	1563.8238	782.4155	1545.8132	773.4102	1546.7972	773.9023	13
6	72.0808	597.3243	299.1658	625.3192	313.1632	607.3086	304.1579	V	1448.7968	724.9021	1430.7863	715.8968	1431.7703	716.3888	12
7	110.0713	734.3832	367.6952	**762.3781**	**381.6927**	744.3675	372.6874	H	***1349.7284***	**675.3678**	1331.7179	666.3626	1332.7019	666.8546	11
8	86.0964	847.4672	424.2373	875.4621	438.2347	857.4516	429.2294	L	***1212.6695***	606.8384	1194.6589	597.8331	**1195.6430**	598.3251	10
9	72.0808	946.5356	473.7715	974.5306	487.7689	956.5200	478.7636	V	***1099.5854***	550.2964	1081.5749	541.2911	**1082.5589**	541.7831	9
10	72.0808	1045.6041	523.3057	1073.5990	537.3031	1055.5884	528.2978	V	***1000.5170***	500.7622	982.5065	491.7569	**983.4905**	492.2489	8
11	101.0709	1173.6626	587.3350	1201.6576	601.3324	1183.6470	592.3271	Q	***901.4486***	451.2279	883.4381	442.2227	**884.4221**	442.7147	7
12	87.0553	1287.7056	644.3564	1315.7005	658.3539	1297.6899	649.3486	N	***773.3900***	387.1987	755.3795	378.1934	**756.3635**	378.6854	6
13	87.0553	1401.7485	701.3779	1429.7434	715.3753	1411.7328	706.3701	N	659.3471	330.1772	641.3365	321.1719	642.3206	321.6639	5
14	102.0550	1530.7911	765.8992	1558.7860	779.8966	1540.7754	770.8914	E	***545.3042***	273.1557	527.2936	264.1504	528.2776	264.6425	4
15	101.0709	1658.8497	829.9285	1686.8446	843.9259	1668.8340	834.9206	Q	***416.2616***	208.6344			399.2350	200.1212	3
16	86.0964	1771.9337	886.4705	1799.9286	900.4680	1781.9181	891.4627	L	288.2030	144.6051			271.1765	136.0919	2
17	129.1135							R	***175.1190***	88.0631			158.0924	79.5498	1

Seq	ya	yb	Seq	ya	yb	Seq	ya	yb
AP	141.1022	169.0972	APE	270.1448	298.1397	APED	385.1718	413.1667
APEDV	484.2402	512.2351	APEDVH	621.2991	649.2940	PE	199.1077	227.1026
PED	314.1347	342.1296	PEDV	413.2031	441.1980	PEDVH	550.2620	578.2569
PEDVHL	663.3461	691.3410	ED	217.0819	245.0768	EDV	316.1503	344.1452
EDVH	453.2092	481.2041	EDVHL	566.2933	594.2882	EDVHLV	665.3617	693.3566
DV	187.1077	215.1026	DVH	324.1666	352.1615	DVHL	437.2507	465.2456
DVHLV	536.3191	564.3140	DVHLVV	635.3875	663.3824	VH	209.1397	237.1346
VHL	322.2238	350.2187	VHLV	421.2922	449.2871	VHLVV	520.3606	548.3555
VHLVVQ	648.4192	676.4141	HL	223.1553	251.1503	HLV	322.2238	350.2187
HLVV	421.2922	449.2871	HLVVQ	549.3507	577.3457	HLVVQN	663.3937	691.3886
LV	185.1648	213.1598	LVV	284.2333	312.2282	LVVQ	412.2918	440.2867
LVVQN	526.3348	554.3297	LVVQNN	640.3777	668.3726	VV	171.1492	199.1441
VVQ	299.2078	327.2027	VVQN	413.2507	441.2456	VVQNN	527.2936	555.2885

H2A.L.1 (Gene :*H2al1n*): **FLREEIHSSR**

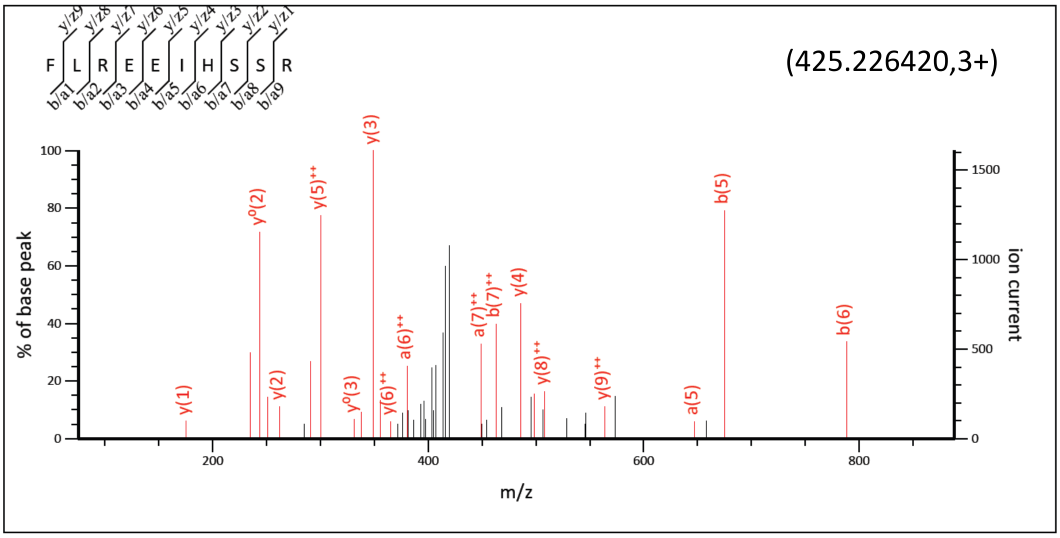

Monoisotopic mass of neutral peptide Mr(calc): 1272.6575
Fixed modifications: Carbamidomethyl (C) (apply to specified residues or termini only)
Ions Score: 41 **Expect:** 0.015
Matches : 33/156 fragment ions using 30 most intense peaks (help)

#	Immon.	a	a^{++}	b	b^{++}	b^{0}	b^{0++}	Seq.	y	y^{++}	y^{0}	y^{0++}	z	z^{++}	#
1	120.0808	120.0808	60.5440	148.0757	74.5415			F							10
2	86.0964	233.1648	117.0861	261.1598	131.0835			L	1126.5963	***563.8018***	1108.5858	554.7965	1109.5698	555.2885	9
3	129.1135	389.2659	195.1366	417.2609	209.1341			R	1013.5123	***507.2598***	995.5017	498.2545	996.4857	**498.7465**	8
4	102.0550	518.3085	259.6579	546.3035	273.6554	528.2929	264.6501	E	857.4112	429.2092	839.4006	420.2039	840.3846	420.6959	7
5	102.0550	647.3511	324.1792	**675.3461**	**338.1767**	657.3355	329.1714	E	728.3686	***364.6879***	710.3580	355.6826	711.3420	**356.1747**	6
6	86.0964	760.4352	**380.7212**	**788.4301**	394.7187	770.4196	385.7134	I	599.3260	***300.1666***	581.3154	291.1613	582.2994	**291.6534**	5
7	110.0713	897.4941	**449.2507**	925.4890	**463.2482**	907.4785	454.2429	H	**486.2419**	***243.6246***	468.2314	234.6193	469.2154	**235.1113**	4
8	60.0444	984.5261	492.7667	1012.5211	**506.7642**	994.5105	497.7589	S	**349.1830**	***175.0951***	331.1724	166.0899	332.1565	166.5819	3
9	60.0444	1071.5582	536.2827	1099.5531	550.2802	1081.5425	541.2749	S	**262.1510**	131.5791	244.1404	122.5738	245.1244	123.0659	2
10	129.1135							R	**175.1190**	88.0631			158.0924	79.5498	1

Seq	ya	yb	Seq	ya	yb	Seq	ya	yb
LR	242.1975	270.1925	LRE	371.2401	399.2350	LREE	500.2827	528.2776
LREEI	613.3668	641.3617	RE	258.1561	286.1510	REE	387.1987	415.1936
REEI	500.2827	528.2776	REEIH	637.3416	665.3365	EE	231.0975	259.0925
EEI	344.1816	372.1765	EEIH	481.2405	509.2354	EEIHS	568.2726	596.2675
EEIHSS	655.3046	683.2995	EI	215.1390	243.1339	EIH	352.1979	380.1928
EIHS	439.2300	467.2249	EIHSS	526.2620	554.2569	IH	223.1553	251.1503
IHS	310.1874	338.1823	IHSS	397.2194	425.2143	HS	197.1033	225.0982
HSS	284.1353	312.1302	SS	147.0764	175.0713			

H2A.L.1 (Gene : *H2al1n*): SGGTSVNEPPEPDDN

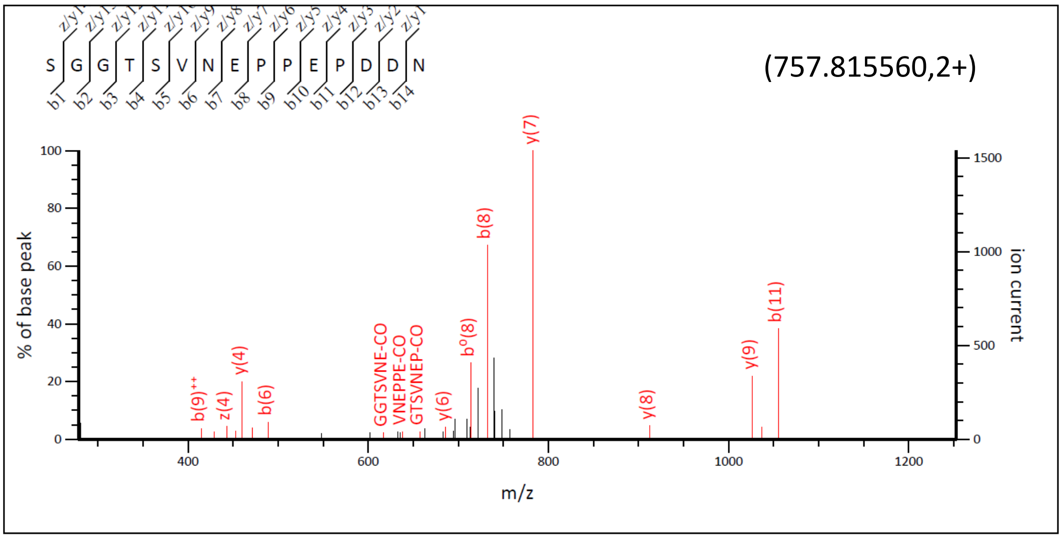

Monoisotopic mass of neutral peptide Mr(calc): 1513.6169
Fixed modifications: Carbamidomethyl (C) (apply to specified residues or termini only)
Ions Score: 28 **Expect:** 0.0073
Matches : 21/286 fragment ions using 31 most intense peaks (help)

#	Immon.	a	a^{++}	b	b^{++}	b^{0}	b^{0++}	Seq.	y	y^{++}	y^{0}	y^{0++}	z	z^{++}	#
1	60.0444	60.0444	30.5258	88.0393	44.5233	70.0287	35.5180	S							15
2	30.0338	117.0659	59.0366	145.0608	73.0340	127.0502	64.0287	G	1427.5921	714.2997	1409.5815	705.2944	1410.5656	705.7864	14
3	30.0338	174.0873	87.5473	202.0822	101.5448	184.0717	92.5395	G	1370.5706	685.7890	1352.5601	676.7837	1353.5441	677.2757	13
4	74.0600	275.1350	138.0711	303.1299	152.0686	285.1193	143.0633	T	1313.5492	657.2782	1295.5386	648.2729	1296.5226	648.7650	12
5	60.0444	362.1670	181.5872	390.1619	195.5846	372.1514	186.5793	S	1212.5015	606.7544	1194.4909	597.7491	1195.4750	598.2411	11
6	72.0808	461.2354	231.1214	489.2304	245.1188	471.2198	236.1135	V	1125.4695	563.2384	1107.4589	554.2331	1108.4429	554.7251	10
7	87.0553	575.2784	288.1428	603.2733	302.1403	585.2627	293.1350	N	1026.4011	513.7042	1008.3905	504.6989	1009.3745	505.1909	9
8	102.0550	704.3210	352.6641	732.3159	366.6616	714.3053	357.6563	E	912.3581	456.6827	894.3476	447.6774	895.3316	448.1694	8
9	70.0651	801.3737	401.1905	829.3686	415.1880	811.3581	406.1827	P	783.3155	392.1614	765.3050	383.1561	766.2890	383.6481	7
10	70.0651	898.4265	449.7169	926.4214	463.7143	908.4108	454.7091	P	686.2628	343.6350	668.2522	334.6297	669.2362	335.1218	6
11	102.0550	1027.4691	514.2382	1055.4640	528.2356	1037.4534	519.2304	E	589.2100	295.1086	571.1994	286.1034	572.1835	286.5954	5
12	70.0651	1124.5218	562.7646	1152.5168	576.7620	1134.5062	567.7567	P	460.1674	230.5873	442.1569	221.5821	443.1409	222.0741	4
13	88.0393	1239.5488	620.2780	1267.5437	634.2755	1249.5331	625.2702	D	363.1147	182.0610	345.1041	173.0557	346.0881	173.5477	3
14	88.0393	1354.5757	677.7915	1382.5706	691.7890	1364.5601	682.7837	D	248.0877	124.5475	230.0771	115.5422	231.0612	116.0342	2
15	87.0553							N	133.0608	67.0340			116.0342	58.5207	1

Seq	ya	yb	Seq	ya	yb	Seq	ya	yb
GG	87.0553	115.0502	GGT	188.1030	216.0979	GGTS	275.1350	303.1299
GGTSV	374.2034	402.1983	GGTSVN	488.2463	516.2413	GGTSVNE	617.2889	645.2838
GT	131.0815	159.0764	GTS	218.1135	246.1084	GTSV	317.1819	345.1769
GTSVN	431.2249	459.2198	GTSVNE	560.2675	588.2624	GTSVNEP	657.3202	685.3151
TS	161.0921	189.0870	TSV	260.1605	288.1554	TSVN	374.2034	402.1983
TSVNE	503.2460	531.2409	TSVNEP	600.2988	628.2937	TSVNEPP	697.3515	725.3464
SV	159.1128	187.1077	SVN	273.1557	301.1506	SVNE	402.1983	430.1932
SVNEP	499.2511	527.2460	SVNEPP	596.3039	624.2988	VN	186.1237	214.1186
VNE	315.1663	343.1612	VNEP	412.2191	440.2140	VNEPP	509.2718	537.2667
VNEPPE	638.3144	666.3093	NE	216.0979	244.0928	NEP	313.1506	341.1456
NEPP	410.2034	438.1983	NEPPE	539.2460	567.2409	NEPPEP	636.2988	664.2937
EP	199.1077	227.1026	EPP	296.1605	324.1554	EPPE	425.2031	453.1980
EPPEP	522.2558	550.2508	EPPEPD	637.2828	665.2777	PP	167.1179	195.1128
PPE	296.1605	324.1554	PPEP	393.2132	421.2082	PPEPD	508.2402	536.2351
PPEPDD	623.2671	651.2620	PE	199.1077	227.1026	PEP	296.1605	324.1554
PEPD	411.1874	439.1823	PEPDD	526.2144	554.2093	EP	199.1077	227.1026
EPD	314.1347	342.1296	EPDD	429.1616	457.1565	PD	185.0921	213.0870
PDD	300.1190	328.1139	DD	203.0662	231.0612			

H2A.L.2 (H2A.Lap3, H2A.B.1): **VVQNNEQLHQLFK**

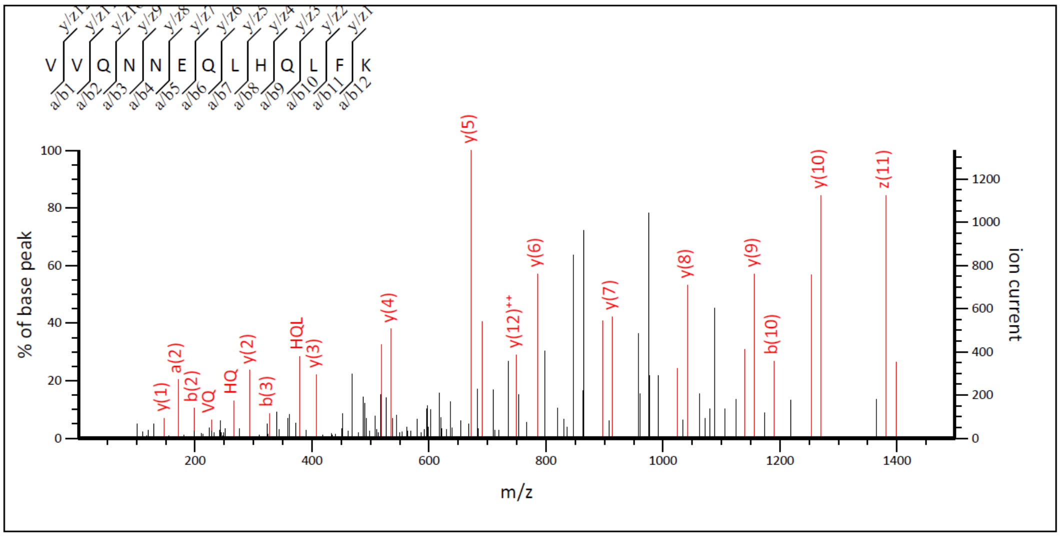

Monoisotopic mass of neutral peptide Mr(calc): 1595.8420
Fixed modifications: Carbamidomethyl (C) (apply to specified residues or termini only)
Ions Score: 85 **Expect:** 1.1e-08
Matches : 28/203 fragment ions using 39 most intense peaks (help)

#	Immon.	a	a++	b	b++	b0	b0++	Seq.	y	y++	y0	y0++	z	z++	#
1	72.0808	72.0808	36.5440	100.0757	50.5415			V							13
2	72.0808	**171.1492**	86.0782	**199.1441**	100.0757			V	1497.7809	**749.3941**	1479.7703	740.3888	1480.7543	740.8808	12
3	101.0709	299.2078	150.1075	**327.2027**	164.1050			Q	***1398.7124***	699.8599	1380.7019	690.8546	**1381.6859**	691.3466	11
4	87.0553	413.2507	207.1290	441.2456	221.1264			N	***1270.6539***	635.8306	1252.6433	626.8253	**1253.6273**	627.3173	10
5	87.0553	527.2936	264.1504	555.2885	278.1479			N	***1156.6109***	578.8091	1138.6004	569.8038	**1139.5844**	570.2958	9
6	102.0550	656.3362	328.6717	684.3311	342.6692	666.3206	333.6639	E	***1042.5680***	521.7876	1024.5574	512.7824	1025.5415	513.2744	8
7	101.0709	784.3948	392.7010	812.3897	406.6985	794.3791	397.6932	Q	***913.5254***	457.2663			**896.4989**	448.7531	7
8	86.0964	897.4789	449.2431	925.4738	463.2405	907.4632	454.2352	L	***785.4668***	393.2371			768.4403	384.7238	6
9	110.0713	1034.5378	517.7725	1062.5327	531.7700	1044.5221	522.7647	H	***672.3828***	336.6950			655.3562	328.1817	5
10	101.0709	1162.5963	581.8018	**1190.5913**	595.7993	1172.5807	586.7940	Q	***535.3239***	268.1656			**518.2973**	259.6523	4
11	86.0964	1275.6804	638.3438	1303.6753	652.3413	1285.6648	643.3360	L	***407.2653***	204.1363			390.2387	195.6230	3
12	120.0808	1422.7488	711.8781	1450.7437	725.8755	1432.7332	716.8702	F	***294.1812***	147.5942			277.1547	139.0810	2
13	101.1073							K	***147.1128***	74.0600			130.0863	65.5468	1

Seq	ya	yb	Seq	ya	yb	Seq	ya	yb
VQ	200.1394	228.1343	VQN	314.1823	342.1772	VQNN	428.2252	456.2201
VQNNE	557.2678	585.2627	VQNNEQ	685.3264	713.3213	QN	215.1139	243.1088
QNN	329.1568	357.1517	QNNE	458.1994	486.1943	QNNEQ	586.2580	614.2529
QNNEQL	699.3420	727.3369	NN	201.0982	229.0931	NNE	330.1408	358.1357
NNEQ	458.1994	486.1943	NNEQL	571.2835	599.2784	NE	216.0979	244.0928
NEQ	344.1565	372.1514	NEQL	457.2405	485.2354	NEQLH	594.2994	622.2944
EQ	230.1135	258.1084	EQL	343.1976	371.1925	EQLH	480.2565	508.2514
EQLHQ	608.3151	636.3100	QL	214.1550	242.1499	QLH	351.2139	379.2088
QLHQ	479.2725	507.2674	QLHQL	592.3566	620.3515	LH	223.1553	251.1503
LHQ	351.2139	379.2088	LHQL	464.2980	492.2929	LHQLF	611.3664	639.3613
HQ	238.1299	266.1248	HQL	351.2139	379.2088	HQLF	498.2823	526.2772
QL	214.1550	242.1499	QLF	361.2234	389.2183	LF	233.1648	261.1598

H2A.L.2 (H2A.Lap3; H2A.B.1): **QGGTSVFEPPEPDDN**

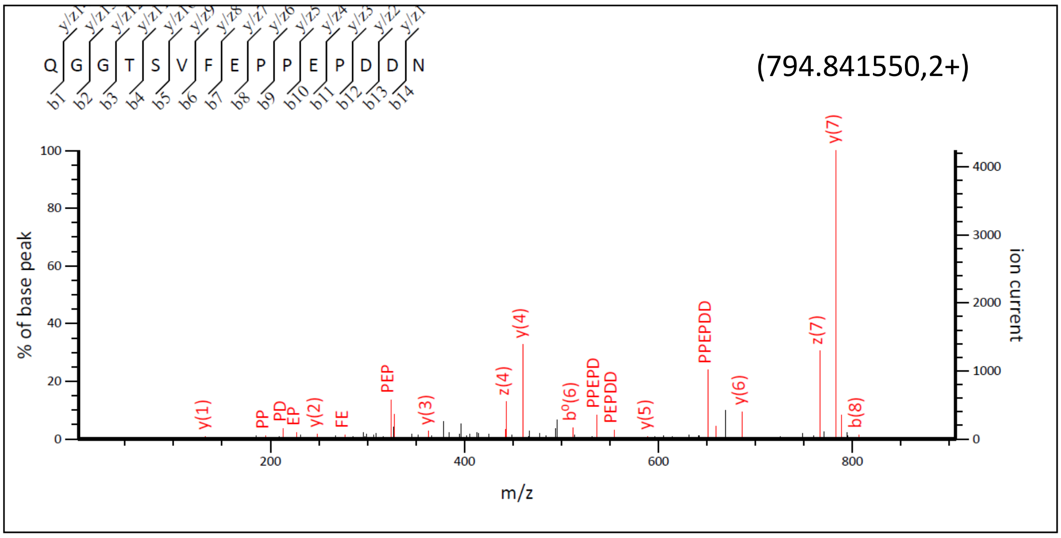

Monoisotopic mass of neutral peptide Mr(calc): 1587.6689
Fixed modifications: Carbamidomethyl (C) (apply to specified residues or termini only)
Ions Score: 39 **Expect:** 0.00048
Matches : 34/278 fragment ions using 43 most intense peaks (help)

#	Immon.	a	a^{++}	b	b^{++}	b^{0}	b^{0++}	Seq.	y	y^{++}	y^{0}	y^{0++}	z	z^{++}	#
1	101.0709	101.0709	51.0391	129.0659	65.0366			Q							15
2	30.0338	158.0924	79.5498	186.0873	93.5473			G	1460.6176	730.8124	1442.6070	721.8072	1443.5910	722.2992	14
3	30.0338	215.1139	108.0606	243.1088	122.0580			G	1403.5961	702.3017	1385.5856	693.2964	1386.5696	693.7884	13
4	74.0600	316.1615	158.5844	344.1565	172.5819	326.1459	163.5766	T	1346.5747	673.7910	1328.5641	664.7857	1329.5481	665.2777	12
5	60.0444	403.1936	202.1004	431.1885	216.0979	413.1779	207.0926	S	1245.5270	623.2671	1227.5164	614.2618	1228.5004	614.7539	11
6	72.0808	502.2620	251.6346	530.2569	265.6321	512.2463	256.6268	V	1158.4950	579.7511	1140.4844	570.7458	1141.4684	571.2378	10
7	120.0808	649.3304	325.1688	677.3253	339.1663	659.3148	330.1610	F	1059.4265	530.2169	1041.4160	521.2116	1042.4000	521.7036	9
8	102.0550	778.3730	389.6901	**806.3679**	403.6876	788.3573	394.6823	E	912.3581	456.6827	894.3476	447.6774	895.3316	448.1694	8
9	70.0651	875.4258	438.2165	903.4207	452.2140	885.4101	443.2087	P	***783.3155***	392.1614	765.3050	383.1561	**766.2890**	383.6481	7
10	70.0651	972.4785	486.7429	1000.4734	500.7404	982.4629	491.7351	P	***686.2628***	343.6350	668.2522	334.6297	669.2362	335.1218	6
11	102.0550	1101.5211	551.2642	1129.5160	565.2617	1111.5055	556.2564	E	***589.2100***	295.1086	571.1994	286.1034	572.1835	286.5954	5
12	70.0651	1198.5739	599.7906	1226.5688	613.7880	1208.5582	604.7828	P	***460.1674***	230.5873	442.1569	221.5821	**443.1409**	222.0741	4
13	88.0393	1313.6008	657.3040	1341.5957	671.3015	1323.5852	662.2962	D	***363.1147***	182.0610	345.1041	173.0557	346.0881	173.5477	3
14	88.0393	1428.6278	714.8175	1456.6227	728.8150	1438.6121	719.8097	D	***248.0877***	124.5475	230.0771	115.5422	231.0612	116.0342	2
15	87.0553							N	***133.0608***	67.0340			116.0342	58.5207	1

Seq	ya	yb	Seq	ya	yb	Seq	ya	yb
GG	87.0553	115.0502	GGT	188.1030	216.0979	GGTS	275.1350	303.1299
GGTSV	374.2034	402.1983	GGTSVF	521.2718	549.2667	GGTSVFE	650.3144	678.3093
GT	131.0815	159.0764	GTS	218.1135	246.1084	GTSV	317.1819	345.1769
GTSVF	464.2504	492.2453	GTSVFE	593.2930	621.2879	GTSVFEP	690.3457	718.3406
TS	161.0921	189.0870	TSV	260.1605	288.1554	TSVF	407.2289	435.2238
TSVFE	536.2715	564.2664	TSVFEP	633.3243	661.3192	SV	159.1128	187.1077
SVF	306.1812	334.1761	SVFE	435.2238	463.2187	SVFEP	532.2766	560.2715
SVFEPP	629.3293	657.3243	VF	219.1492	247.1441	VFE	348.1918	376.1867
VFEP	445.2445	473.2395	VFEPP	542.2973	570.2922	VFEPPE	671.3399	699.3348
FE	249.1234	277.1183	FEP	346.1761	374.1710	FEPP	443.2289	471.2238
FEPPE	572.2715	600.2664	FEPPEP	669.3243	697.3192	EP	199.1077	227.1026
EPP	296.1605	324.1554	EPPE	425.2031	453.1980	EPPEP	522.2558	550.2508
EPPEPD	637.2828	665.2777	PP	167.1179	195.1128	PPE	296.1605	324.1554
PPEP	393.2132	421.2082	PPEPD	508.2402	536.2351	PPEPDD	623.2671	651.2620
PE	199.1077	227.1026	PEP	296.1605	324.1554	PEPD	411.1874	439.1823
PEPDD	526.2144	554.2093	EP	199.1077	227.1026	EPD	314.1347	342.1296
EPDD	429.1616	457.1565	PD	185.0921	213.0870	PDD	300.1190	328.1139
DD	203.0662	231.0612						

H2A.L.2 (H2A.Lap3; H2A.B.1): **IAPEHVCR**

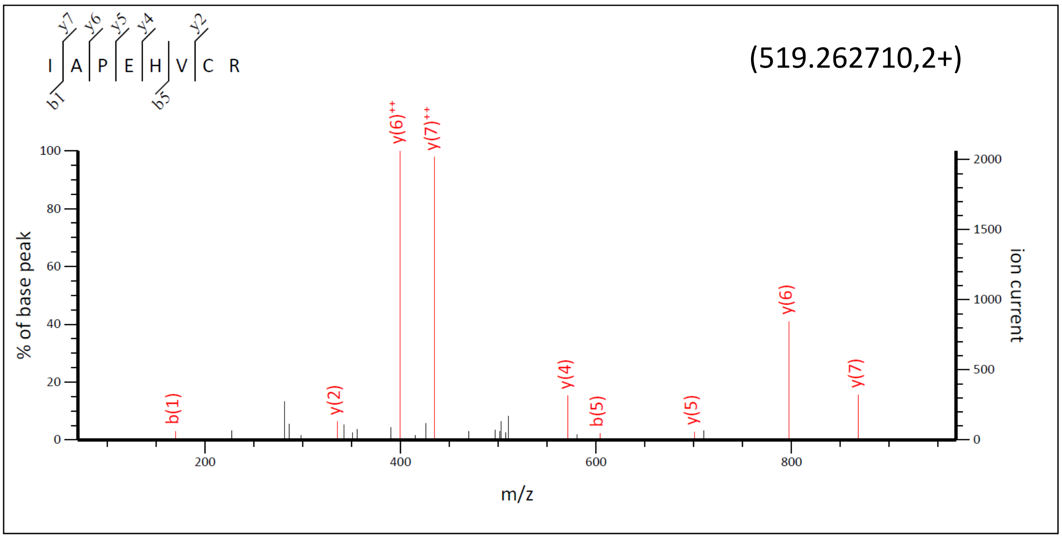


```
Monoisotopic mass of neutral peptide Mr(calc): 1036.5124
Fixed modifications: Carbamidomethyl (C),Propionyl (N-term) (apply to specified residues or termini only)
Ions Score: 47  Expect: 0.0024
Matches : 11/56 fragment ions using 14 most intense peaks   (help)
```

#	b	b++	b0	b0++	Seq.	y	y++	y*	y*++	y0	y0++	#
1	**170.1176**	85.5624			I							8
2	241.1547	121.0810			A	***868.4094***	**434.7083**	851.3828	426.1951	850.3988	425.7031	7
3	338.2074	169.6074			P	***797.3723***	**399.1898**	780.3457	390.6765	779.3617	390.1845	6
4	467.2500	234.1287	449.2395	225.1234	E	***700.3195***	350.6634	683.2930	342.1501	682.3090	341.6581	5
5	**604.3089**	302.6581	586.2984	293.6528	H	***571.2769***	286.1421	554.2504	277.6288			4
6	703.3774	352.1923	685.3668	343.1870	V	***434.2180***	217.6126	417.1915	209.0994			3
7	863.4080	432.2076	845.3974	423.2024	C	***335.1496***	168.0784	318.1231	159.5652			2
8					R	175.1190	88.0631	158.0924	79.5498			1

H2B.L.2: **NIHVGLSLSQR**

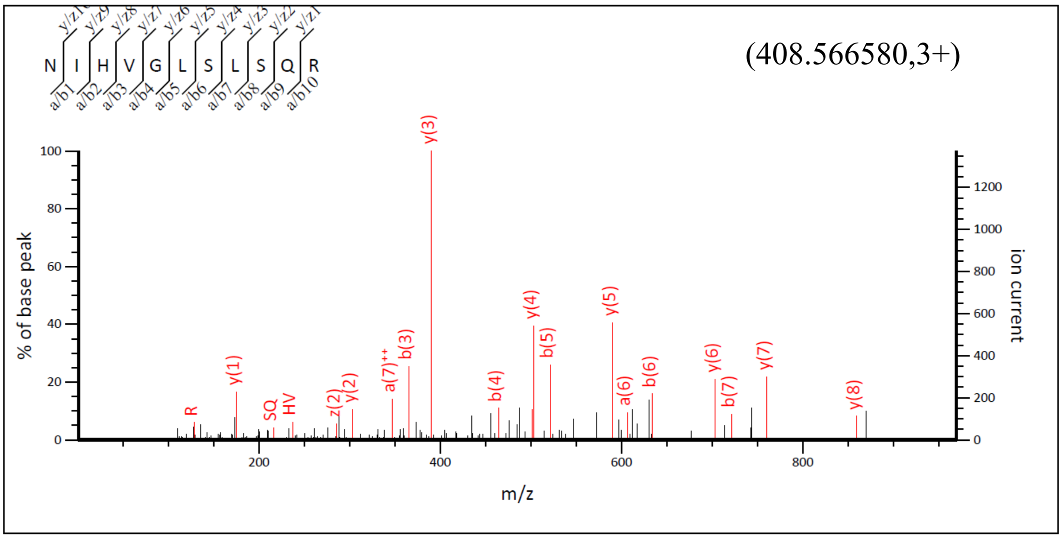

Monoisotopic mass of neutral peptide Mr(calc): 1222.6782
Fixed modifications: Carbamidomethyl (C) (apply to specified residues or termini only)
Ions Score: 60 **Expect:** 3e-05
Matches : 22/180 fragment ions using 30 most intense peaks (help)

#	Immon.	a	a^{++}	b	b^{++}	b^{0}	b^{0++}	Seq.	y	y^{++}	y^{0}	y^{0++}	z	z^{++}	#
1	87.0553	87.0553	44.0313	115.0502	58.0287			N							11
2	86.0964	200.1394	100.5733	228.1343	114.5708			I	1109.6426	555.3249	1091.6320	546.3196	1092.6160	546.8116	10
3	110.0713	337.1983	169.1028	**365.1932**	183.1002			H	996.5585	498.7829	978.5479	489.7776	979.5320	490.2696	9
4	72.0808	436.2667	218.6370	**464.2616**	232.6344			V	***859.4996***	430.2534	841.4890	421.2482	842.4730	421.7402	8
5	30.0338	493.2881	247.1477	**521.2831**	261.1452			G	***760.4312***	380.7192	742.4206	371.7139	743.4046	372.2060	7
6	86.0964	**606.3722**	303.6897	**634.3671**	317.6872			L	***703.4097***	352.2085	685.3991	343.2032	686.3832	343.6952	6
7	60.0444	693.4042	**347.2058**	**721.3991**	361.2032	703.3886	352.1979	S	***590.3256***	295.6665	572.3151	286.6612	573.2991	287.1532	5
8	86.0964	806.4883	403.7478	834.4832	417.7452	816.4726	408.7400	L	***503.2936***	252.1504	485.2831	243.1452	486.2671	243.6372	4
9	60.0444	893.5203	447.2638	921.5152	461.2613	903.5047	452.2560	S	***390.2096***	195.6084	372.1990	186.6031	373.1830	187.0951	3
10	101.0709	1021.5789	511.2931	1049.5738	525.2905	1031.5633	516.2853	Q	***303.1775***	152.0924			**286.1510**	143.5791	2
11	129.1135							R	***175.1190***	88.0631			158.0924	79.5498	1

Seq	ya	yb	Seq	ya	yb	Seq	ya	yb
**IH**	223.1553	251.1503	**IHV**	322.2238	350.2187	**IHVG**	379.2452	407.2401
**IHVGL**	492.3293	520.3242	**IHVGLS**	579.3613	607.3562	**IHVGLSL**	692.4454	720.4403
**HV**	209.1397	237.1346	**HVG**	266.1612	294.1561	**HVGL**	379.2452	407.2401
**HVGLS**	466.2772	494.2722	**HVGLSL**	579.3613	607.3562	**HVGLSLS**	666.3933	694.3883
**VG**	129.1022	157.0972	**VGL**	242.1863	270.1812	**VGLS**	329.2183	357.2132
**VGLSL**	442.3024	470.2973	**VGLSLS**	529.3344	557.3293	**VGLSLSQ**	657.3930	685.3879
**GL**	143.1179	171.1128	**GLS**	230.1499	258.1448	**GLSL**	343.2340	371.2289
**GLSLS**	430.2660	458.2609	**GLSLSQ**	558.3246	586.3195	**LS**	173.1285	201.1234
**LSL**	286.2125	314.2074	**LSLS**	373.2445	401.2395	**LSLSQ**	501.3031	529.2980
**SL**	173.1285	201.1234	**SLS**	260.1605	288.1554	**SLSQ**	388.2191	416.2140
**LS**	173.1285	201.1234	**LSQ**	301.1870	329.1819	**SQ**	188.1030	216.0979

H2B.L.2: **IASEASFLAR**

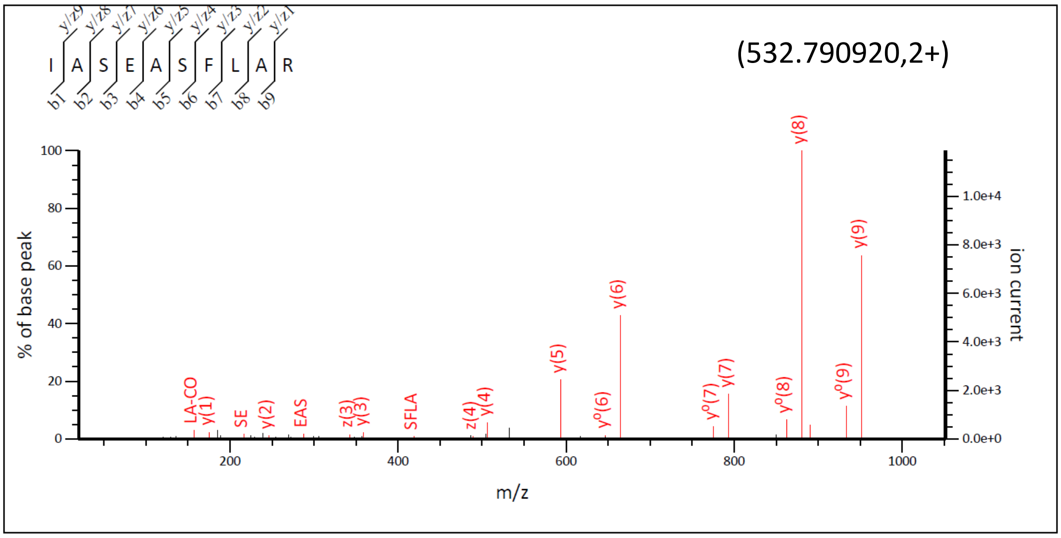

Monoisotopic mass of neutral peptide Mr(calc): 1063.5662
Fixed modifications: Carbamidomethyl (C) (apply to specified residues or termini only)
Ions Score: 83 **Expect:** 5.8e-07
Matches : 26/158 fragment ions using 30 most intense peaks (help)

#	Immon.	a	a^{++}	b	b^{++}	b^{0}	b^{0++}	Seq.	y	y^{++}	y^{0}	y^{0++}	z	z^{++}	#
1	86.0964	86.0964	43.5519	114.0913	57.5493			I							10
2	44.0495	**157.1335**	79.0704	**185.1285**	93.0679			A	***951.4894***	476.2483	933.4789	467.2431	934.4629	467.7351	9
3	60.0444	244.1656	122.5864	272.1605	136.5839	254.1499	127.5786	S	***880.4523***	440.7298	862.4417	431.7245	863.4258	432.2165	8
4	102.0550	373.2082	187.1077	401.2031	201.1052	383.1925	192.0999	E	***793.4203***	397.2138	775.4097	388.2085	776.3937	388.7005	7
5	44.0495	444.2453	222.6263	472.2402	236.6237	454.2296	227.6184	A	***664.3777***	332.6925	646.3671	323.6872	647.3511	324.1792	6
6	60.0444	531.2773	266.1423	559.2722	280.1397	541.2617	271.1345	S	***593.3406***	297.1739	575.3300	288.1686	576.3140	288.6606	5
7	120.0808	678.3457	339.6765	706.3406	353.6740	688.3301	344.6687	F	***506.3085***	253.6579			**489.2820**	245.1446	4
8	86.0964	791.4298	396.2185	819.4247	410.2160	801.4141	401.2107	L	***359.2401***	180.1237			**342.2136**	171.6104	3
9	44.0495	862.4669	431.7371	**890.4618**	445.7345	872.4512	436.7293	A	***246.1561***	123.5817			229.1295	115.0684	2
10	129.1135							R	***175.1190***	88.0631			158.0924	79.5498	1

Seq	ya	yb	Seq	ya	yb	Seq	ya	yb
AS	131.0815	159.0764	ASE	260.1241	288.1190	ASEA	331.1612	359.1561
ASEAS	418.1932	446.1882	ASEASF	565.2617	593.2566	ASEASFL	678.3457	706.3406
SE	189.0870	217.0819	SEA	260.1241	288.1190	SEAS	347.1561	375.1510
SEASF	494.2245	522.2195	SEASFL	607.3086	635.3035	SEASFLA	678.3457	706.3406
EA	173.0921	201.0870	EAS	260.1241	288.1190	EASF	407.1925	435.1874
EASFL	520.2766	548.2715	EASFLA	591.3137	619.3086	AS	131.0815	159.0764
ASF	278.1499	306.1448	ASFL	391.2340	419.2289	ASFLA	462.2711	490.2660
SF	207.1128	235.1077	SFL	320.1969	348.1918	SFLA	391.2340	419.2289
FL	233.1648	261.1598	FLA	304.2020	332.1969	LA	157.1335	185.1285

H2B.L.2: **NSFAIYFPK**

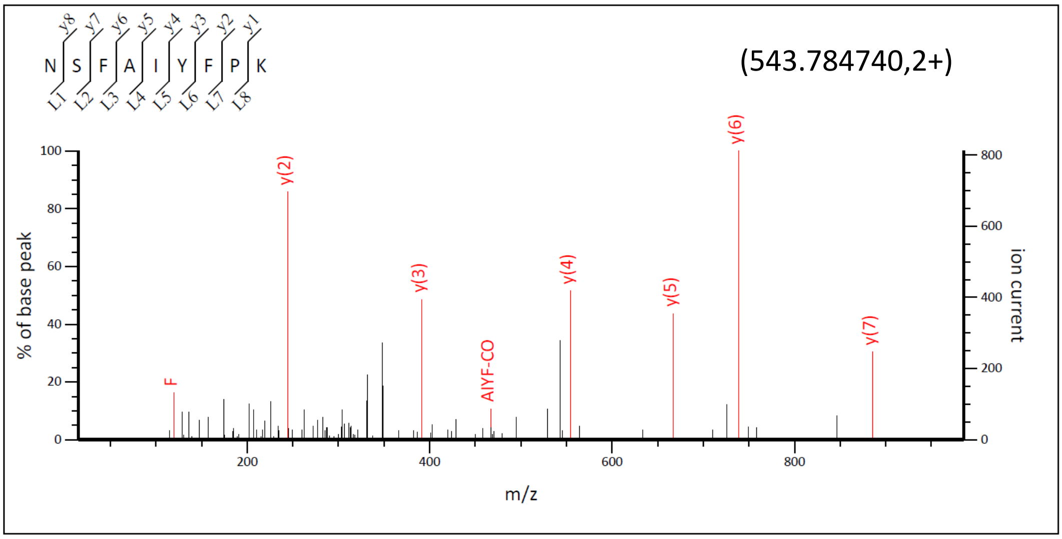

Monoisotopic mass of neutral peptide Mr(calc): 1085.5546
Fixed modifications: Carbamidomethyl (C) (apply to specified residues or termini only)
Ions Score: 64 **Expect:** 4.1e-06
Matches : 12/125 fragment ions using 8 most intense peaks (help)

#	Immon.	a	a^{++}	b	b^{++}	b^{0}	b^{0++}	Seq.	y	y^{++}	y^{0}	y^{0++}	z	z^{++}	#
1	87.0553	87.0553	44.0313	115.0502	58.0287			N							9
2	60.0444	174.0873	87.5473	202.0822	101.5448	184.0717	92.5395	S	972.5189	486.7631	954.5084	477.7578	955.4924	478.2498	8
3	120.0808	321.1557	161.0815	349.1506	175.0790	331.1401	166.0737	F	***885.4869***	443.2471			868.4604	434.7338	7
4	44.0495	392.1928	196.6001	420.1878	210.5975	402.1772	201.5922	A	***738.4185***	369.7129			721.3919	361.1996	6
5	86.0964	505.2769	253.1421	533.2718	267.1395	515.2613	258.1343	I	***667.3814***	334.1943			650.3548	325.6811	5
6	136.0757	668.3402	334.6738	696.3352	348.6712	678.3246	339.6659	Y	***554.2973***	277.6523			537.2708	269.1390	4
7	120.0808	815.4087	408.2080	843.4036	422.2054	825.3930	413.2001	F	***391.2340***	196.1206			374.2074	187.6074	3
8	70.0651	912.4614	456.7343	940.4563	470.7318	922.4458	461.7265	P	***244.1656***	122.5864			227.1390	114.0731	2
9	101.1073							K	147.1128	74.0600			130.0863	65.5468	1

Seq	ya	yb	Seq	ya	yb	Seq	ya	yb
SF	207.1128	235.1077	SFA	278.1499	306.1448	SFAI	391.2340	419.2289
SFAIY	554.2973	582.2922	FA	191.1179	219.1128	FAI	304.2020	332.1969
FAIY	467.2653	495.2602	FAIYF	614.3337	642.3286	AI	157.1335	185.1285
AIY	320.1969	348.1918	AIYF	467.2653	495.2602	AIYFP	564.3180	592.3130
IY	249.1598	277.1547	IYF	396.2282	424.2231	IYFP	493.2809	521.2758
YF	283.1441	311.1390	YFP	380.1969	408.1918	FP	217.1335	245.1285

H2B.L.2: **SVNILDSFVK**

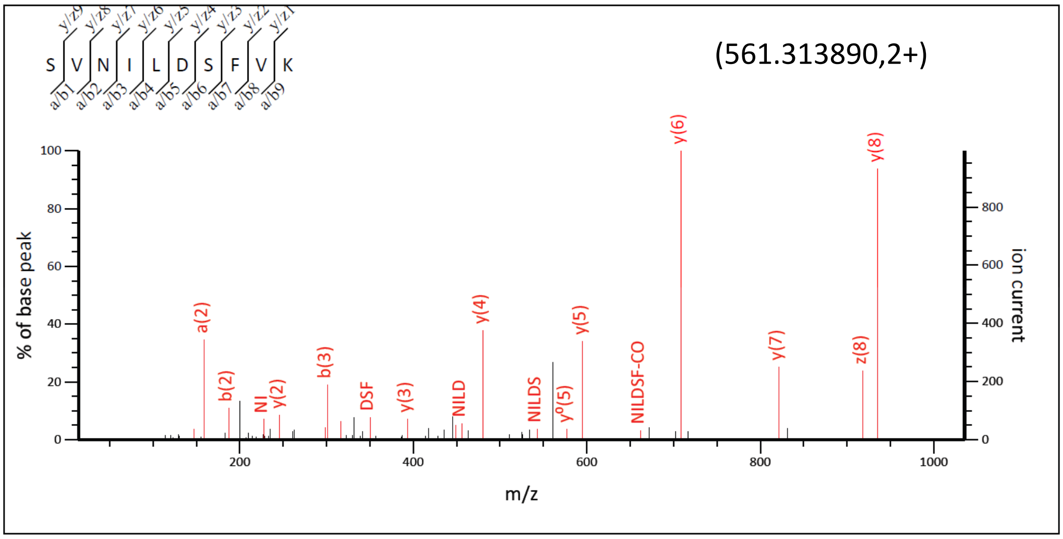

Monoisotopic mass of neutral peptide Mr(calc): 1120.6128
Fixed modifications: Carbamidomethyl (C) (apply to specified residues or termini only)
Ions Score: 67 **Expect:** 1.5e-06
Matches : 21/162 fragment ions using 29 most intense peaks (help)

#	Immon.	a	a++	b	b++	b0	b0++	Seq.	y	y++	y0	y0++	z	z++	#
1	60.0444	60.0444	30.5258	88.0393	44.5233	70.0287	35.5180	S							10
2	72.0808	**159.1128**	80.0600	**187.1077**	94.0575	169.0972	85.0522	V	1034.5881	517.7977	1016.5775	508.7924	1017.5615	509.2844	9
3	87.0553	273.1557	137.0815	**301.1506**	151.0790	283.1401	142.0737	N	***935.5197***	468.2635	917.5091	459.2582	**918.4931**	459.7502	8
4	86.0964	386.2398	193.6235	414.2347	207.6210	396.2241	198.6157	I	***821.4767***	411.2420	803.4662	402.2367	804.4502	402.7287	7
5	86.0964	499.3239	250.1656	527.3188	264.1630	509.3082	255.1577	L	***708.3927***	354.7000	690.3821	345.6947	691.3661	346.1867	6
6	88.0393	614.3508	307.6790	642.3457	321.6765	624.3352	312.6712	D	***595.3086***	**298.1579**	577.2980	289.1527	578.2821	289.6447	5
7	60.0444	701.3828	351.1951	729.3777	365.1925	711.3672	356.1872	S	***480.2817***	240.6445	462.2711	231.6392	463.2551	232.1312	4
8	120.0808	848.4512	424.7293	876.4462	438.7267	858.4356	429.7214	F	***393.2496***	197.1285			376.2231	188.6152	3
9	72.0808	947.5197	474.2635	975.5146	488.2609	957.5040	479.2556	V	***246.1812***	123.5942			229.1547	115.0810	2
10	101.1073							K	***147.1128***	74.0600			130.0863	65.5468	1

Seq	ya	yb	Seq	ya	yb	Seq	ya	yb
VN	186.1237	214.1186	VNI	299.2078	327.2027	VNIL	412.2918	440.2867
VNILD	527.3188	555.3137	VNILDS	614.3508	642.3457	NI	200.1394	228.1343
NIL	313.2234	341.2183	NILD	428.2504	456.2453	NILDS	515.2824	543.2773
NILDSF	662.3508	690.3457	IL	199.1805	227.1754	ILD	314.2074	342.2023
ILDS	401.2395	429.2344	ILDSF	548.3079	576.3028	ILDSFV	647.3763	675.3712
LD	201.1234	229.1183	LDS	288.1554	316.1503	LDSF	435.2238	463.2187
LDSFV	534.2922	562.2871	DS	175.0713	203.0662	DSF	322.1397	350.1347
DSFV	421.2082	449.2031	SF	207.1128	235.1077	SFV	306.1812	334.1761
FV	219.1492	247.1441						

H3.3 (Gene: *Gm10257*): **K(ac)SVPSTGGVK(pr)K(pr)PHR**

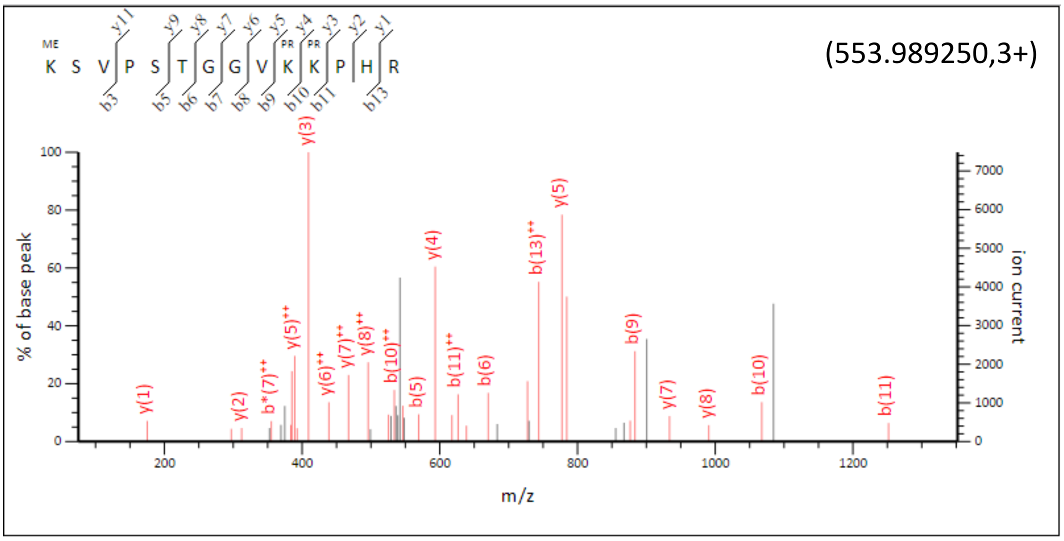


```
Monoisotopic mass of neutral peptide Mr(calc): 1658.9468
Fixed modifications: Carbamidomethyl (C),Propionyl (N-term) (apply to specified residues or termini only)
Variable modifications:
K1      : Methyl (K)
K10     : Propionyl (K)
K11     : Propionyl (K)
Ions Score: 64  Expect: 0.00017
Matches : 35/138 fragment ions using 42 most intense peaks   (help)
```

#	b	b^{++}	b*	b^{*++}	b^0	b^{0++}	Seq.	y	y^{++}	y*	y^{*++}	y^0	y^{0++}	#
1	199.1441	100.0757	182.1176	91.5624			K							14
2	286.1761	143.5917	269.1496	135.0784	268.1656	134.5864	S	1461.8172	731.4123	1444.7907	722.8990	1443.8067	722.4070	13
3	385.2445	193.1259	368.2180	184.6126	367.2340	184.1206	V	1374.7852	687.8962	1357.7587	679.3830	1356.7746	678.8910	12
4	482.2973	241.6523	465.2708	233.1390	464.2867	232.6470	P	1275.7168	638.3620	1258.6902	629.8488	1257.7062	629.3568	11
5	569.3293	285.1683	552.3028	276.6550	551.3188	276.1630	S	1178.6640	589.8357	1161.6375	581.3224	1160.6535	580.8304	10
6	670.3770	335.6921	653.3505	327.1789	652.3665	326.6869	T	1091.6320	546.3196	1074.6055	537.8064	1073.6214	537.3144	9
7	727.3985	364.2029	710.3719	355.6896	709.3879	355.1976	G	990.5843	495.7958	973.5578	487.2825			8
8	784.4199	392.7136	767.3934	384.2003	766.4094	383.7083	G	933.5629	467.2851	916.5363	458.7718			7
9	883.4884	442.2478	866.4618	433.7345	865.4778	433.2425	V	876.5414	438.7743	859.5148	430.2611			6
10	1067.6095	534.3084	1050.5830	525.7951	1049.5990	525.3031	K	777.4730	389.2401	760.4464	380.7269			5
11	1251.7307	626.3690	1234.7042	617.8557	1233.7202	617.3637	K	593.3518	297.1795	576.3253	288.6663			4
12	1348.7835	674.8954	1331.7569	666.3821	1330.7729	665.8901	P	409.2306	205.1190	392.2041	196.6057			3
13	1485.8424	743.4248	1468.8158	734.9116	1467.8318	734.4196	H	312.1779	156.5926	295.1513	148.0793			2
14							R	175.1190	88.0631	158.0924	79.5498			1

H3.3 (Gene: *Gm6421*): **RYQK(ac)ATELLIR**

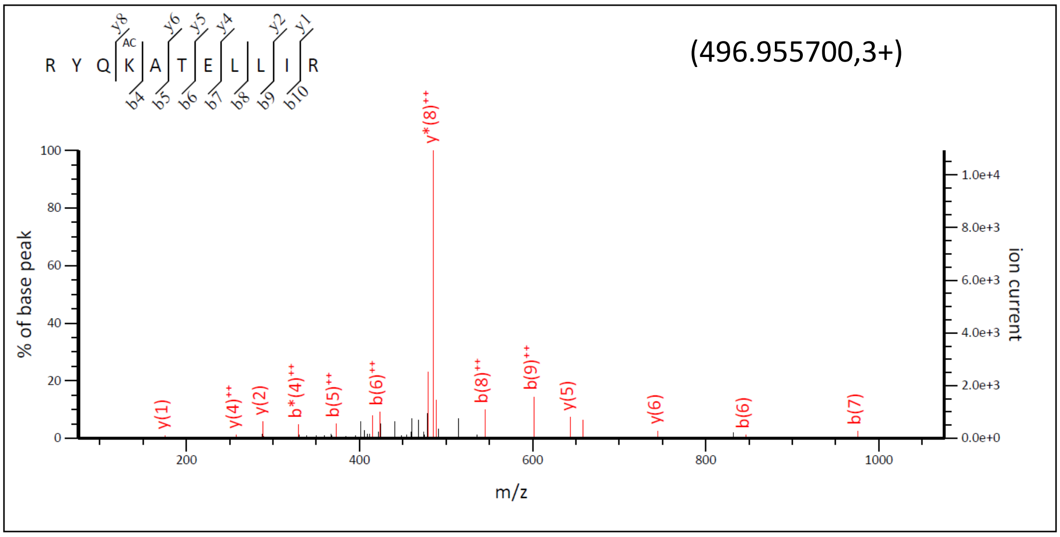

Monoisotopic mass of neutral peptide Mr(calc): 1487.8460
Fixed modifications: Carbamidomethyl (C),Propionyl (N-term) (apply to specified residues or termini only)
Variable modifications:
K4 : Acetyl (K)
Ions Score: 46 **Expect:** 0.013
Matches : 20/102 fragment ions using 21 most intense peaks (help)

#	b	b^{++}	b^{*}	b^{*++}	b^{0}	b^{0++}	Seq.	y	y^{++}	y^{*}	y^{*++}	y^{0}	y^{0++}	#
1	213.1346	107.0709	196.1081	98.5577			R							11
2	376.1979	188.6026	359.1714	180.0893			Y	1276.7260	638.8666	1259.6994	630.3533	1258.7154	629.8613	10
3	504.2565	252.6319	487.2300	244.1186			Q	1113.6626	557.3350	1096.6361	548.8217	1095.6521	548.3297	9
4	674.3620	337.6847	657.3355	329.1714			K	985.6041	493.3057	968.5775	484.7924	967.5935	484.3004	8
5	745.3992	*373.2032*	728.3726	364.6899			A	815.4985	408.2529	798.4720	399.7396	797.4880	399.2476	7
6	**846.4468**	*423.7271*	829.4203	415.2138	828.4363	414.7218	T	**744.4614**	**372.7343**	727.4349	364.2211	726.4509	363.7291	6
7	**975.4894**	*488.2483*	958.4629	479.7351	957.4789	479.2431	E	**643.4137**	322.2105	626.3872	313.6972	625.4032	313.2052	5
8	1088.5735	*544.7904*	1071.5469	536.2771	1070.5629	535.7851	L	514.3711	**257.6892**	497.3446	249.1759			4
9	1201.6576	*601.3324*	1184.6310	592.8191	1183.6470	592.3271	L	401.2871	201.1472	384.2605	192.6339			3
10	1314.7416	*657.8744*	1297.7151	649.3612	1296.7310	648.8692	I	**288.2030**	144.6051	271.1765	136.0919			2
11							R	**175.1190**	88.0631	158.0924	79.5498			1

H1.0 (H10): **YSDMIVAAIQAEK**

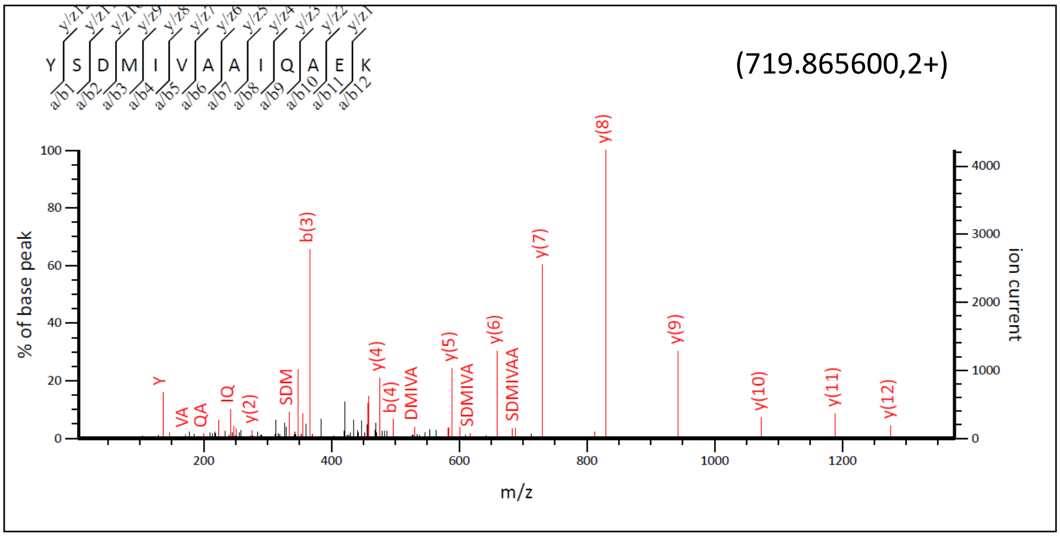

Monoisotopic mass of neutral peptide Mr(calc): 1437.7173
Fixed modifications: Carbamidomethyl (C) (apply to specified residues or termini only)
Ions Score: 135 **Expect:** 8.2e-13
Matches : 38/241 fragment ions using 38 most intense peaks (help)

#	Immon.	a	a^{++}	b	b^{++}	b^{0}	b^{0++}	Seq.	y	y^{++}	y^{0}	y^{0++}	z	z^{++}	#
1	136.0757	**136.0757**	68.5415	164.0706	82.5389			Y							13
2	60.0444	**223.1077**	112.0575	**251.1026**	126.0550	233.0921	117.0497	S	***1275.6613***	638.3343	1257.6508	629.3290	1258.6348	629.8210	12
3	88.0393	338.1347	169.5710	**366.1296**	183.5684	348.1190	174.5631	D	***1188.6293***	594.8183	1170.6187	585.8130	1171.6027	586.3050	11
4	104.0528	469.1751	235.0912	**497.1701**	249.0887	479.1595	240.0834	M	***1073.6023***	537.3048	1055.5918	528.2995	1056.5758	528.7915	10
5	86.0964	**582.2592**	291.6332	610.2541	305.6307	592.2436	296.6254	I	***942.5619***	471.7846	924.5513	462.7793	925.5353	463.2713	9
6	72.0808	681.3276	341.1675	709.3225	355.1649	691.3120	346.1596	V	***829.4778***	415.2425	811.4672	406.2373	**812.4512**	406.7293	8
7	44.0495	752.3647	376.6860	780.3597	390.6835	762.3491	381.6782	A	***730.4094***	365.7083	712.3988	356.7030	713.3828	357.1951	7
8	44.0495	823.4019	412.2046	851.3968	426.2020	833.3862	417.1967	A	***659.3723***	330.1898	641.3617	321.1845	**642.3457**	321.6765	6
9	86.0964	936.4859	468.7466	964.4808	482.7441	946.4703	473.7388	I	***588.3352***	294.6712	570.3246	285.6659	571.3086	286.1579	5
10	101.0709	1064.5445	532.7759	1092.5394	546.7733	1074.5288	537.7681	Q	***475.2511***	238.1292	457.2405	229.1239	**458.2245**	229.6159	4
11	44.0495	1135.5816	568.2944	1163.5765	582.2919	1145.5660	573.2866	A	***347.1925***	174.0999	329.1819	165.0946	330.1660	165.5866	3
12	102.0550	1264.6242	632.8157	1292.6191	646.8132	1274.6086	637.8079	E	***276.1554***	138.5813	258.1448	129.5761	259.1288	130.0681	2
13	101.1073							K	***147.1128***	74.0600			130.0863	65.5468	1

Seq	ya	yb	Seq	ya	yb	Seq	ya	yb
SD	175.0713	203.0662	SDM	306.1118	334.1067	SDMI	419.1959	447.1908
SDMIV	518.2643	546.2592	SDMIVA	589.3014	617.2963	SDMIVAA	660.3385	688.3334
DM	219.0798	247.0747	DMI	332.1639	360.1588	DMIV	431.2323	459.2272
DMIVA	502.2694	530.2643	DMIVAA	573.3065	601.3014	DMIVAAI	686.3906	714.3855
MI	217.1369	245.1318	MIV	316.2053	344.2002	MIVA	387.2424	415.2374
MIVAA	458.2796	486.2745	MIVAAI	571.3636	599.3585	MIVAAIQ	699.4222	727.4171
IV	185.1648	213.1598	IVA	256.2020	284.1969	IVAA	327.2391	355.2340
IVAAI	440.3231	468.3180	IVAAIQ	568.3817	596.3766	IVAAIQA	639.4188	667.4137
VA	143.1179	171.1128	VAA	214.1550	242.1499	VAAI	327.2391	355.2340
VAAIQ	455.2976	483.2926	VAAIQA	526.3348	554.3297	VAAIQAE	655.3774	683.3723
AA	115.0866	143.0815	AAI	228.1707	256.1656	AAIQ	356.2292	384.2241
AAIQA	427.2663	455.2613	AAIQAE	556.3089	584.3039	AI	157.1335	185.1285
AIQ	285.1921	313.1870	AIQA	356.2292	384.2241	AIQAE	485.2718	513.2667
IQ	214.1550	242.1499	IQA	285.1921	313.1870	IQAE	414.2347	442.2296
QA	172.1081	200.1030	QAE	301.1506	329.1456	AE	173.0921	201.0870

H1.0 (H1°): LVTTGVLK

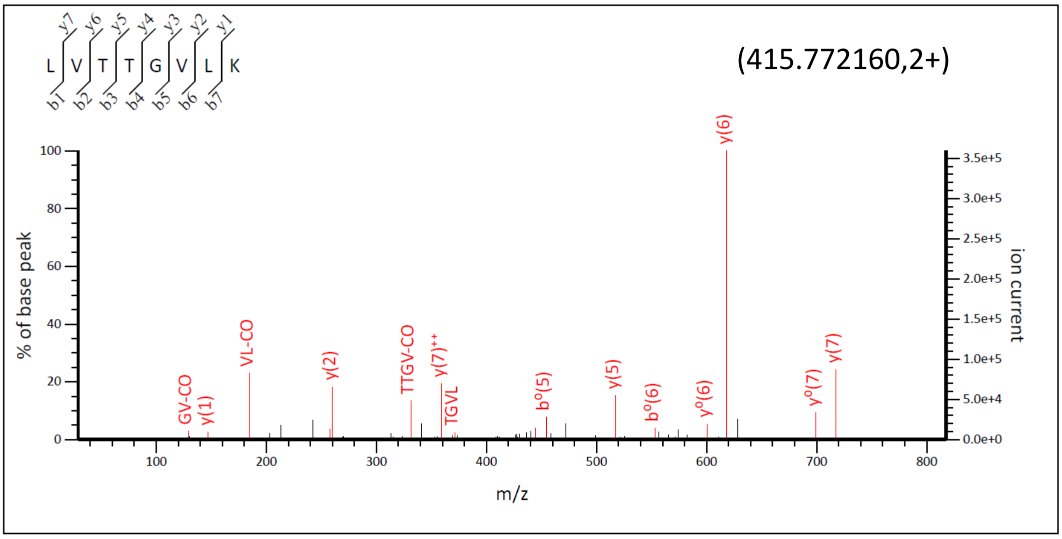

Label all possible matches ○ Label matches used for scoring ◉

Monoisotopic mass of neutral peptide Mr(calc): 829.5273
Fixed modifications: Carbamidomethyl (C) (apply to specified residues or termini only)
Ions Score: 46 **Expect:** 0.0035
Matches : 26/110 fragment ions using 22 most intense peaks (help)

#	Immon.	a	a^{++}	b	b^{++}	b^0	b^{0++}	Seq.	y	y^{++}	y^0	y^{0++}	z	z^{++}	#
1	86.0964	86.0964	43.5519	114.0913	57.5493			L							8
2	72.0808	**185.1648**	93.0861	**213.1598**	107.0835			V	***717.4505***	**359.2289**	699.4400	350.2236	700.4240	350.7156	7
3	74.0600	286.2125	143.6099	314.2074	157.6074	296.1969	148.6021	T	***618.3821***	309.6947	600.3715	300.6894	601.3556	301.1814	6
4	74.0600	387.2602	194.1337	415.2551	208.1312	397.2445	199.1259	T	***517.3344***	259.1709	499.3239	250.1656	500.3079	250.6576	5
5	30.0338	**444.2817**	222.6445	**472.2766**	236.6419	454.2660	227.6366	G	416.2867	208.6470			399.2602	200.1337	4
6	72.0808	543.3501	272.1787	571.3450	286.1761	553.3344	277.1709	V	359.2653	180.1363			342.2387	171.6230	3
7	86.0964	656.4341	328.7207	684.4291	342.7182	666.4185	333.7129	L	***260.1969***	130.6021			243.1703	122.0888	2
8	101.1073							K	***147.1128***	74.0600			130.0863	65.5468	1

Seq	ya	yb	Seq	ya	yb	Seq	ya	yb
VT	173.1285	201.1234	VTT	274.1761	302.1710	VTTG	331.1976	359.1925
VTTGV	430.2660	458.2609	VTTGVL	543.3501	571.3450	TT	175.1077	203.1026
TTG	232.1292	260.1241	TTGV	331.1976	359.1925	TTGVL	444.2817	472.2766
TG	131.0815	159.0764	TGV	230.1499	258.1448	TGVL	343.2340	371.2289
GV	129.1022	157.0972	GVL	242.1863	270.1812	VL	185.1648	213.1598

H1.0 (H1°): **RLVTTGVLK**

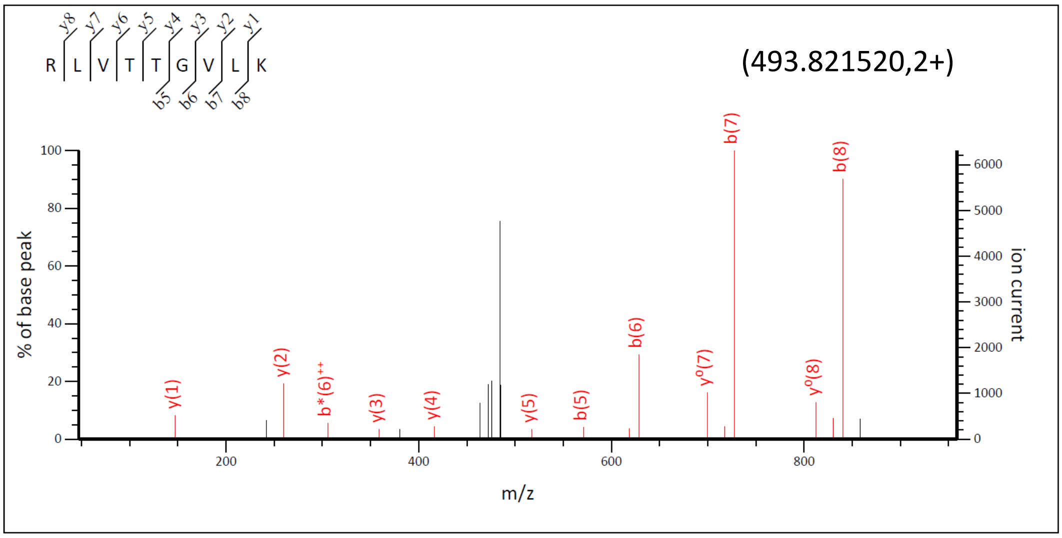

Monoisotopic mass of neutral peptide Mr(calc): 985.6284
Fixed modifications: Carbamidomethyl (C) (apply to specified residues or termini only)
Ions Score: 79 **Expect:** 3.6e-06
Matches : 18/82 fragment ions using 23 most intense peaks (help)

#	b	b^{++}	b*	b^{*++}	b^0	b^{0++}	Seq.	y	y^{++}	y*	y^{*++}	y^0	y^{0++}	#
1	157.1084	79.0578	140.0818	70.5446			R							9
2	270.1925	135.5999	253.1659	127.0866			L	*830.5346*	**415.7709**	813.5080	407.2577	812.5240	406.7656	8
3	369.2609	185.1341	352.2343	176.6208			V	*717.4505*	**359.2289**	700.4240	350.7156	699.4400	350.2236	7
4	470.3085	235.6579	453.2820	227.1446	452.2980	226.6526	T	*618.3821*	309.6947	601.3556	301.1814	600.3715	300.6894	6
5	**571.3562**	286.1817	554.3297	277.6685	553.3457	277.1765	T	*517.3344*	259.1709	500.3079	250.6576	499.3239	250.1656	5
6	**628.3777**	314.6925	611.3511	306.1792	610.3671	305.6872	G	*416.2867*	208.6470	399.2602	200.1337			4
7	**727.4461**	364.2267	710.4196	355.7134	709.4355	355.2214	V	*359.2653*	180.1363	342.2387	171.6230			3
8	**840.5302**	420.7687	823.5036	412.2554	822.5196	411.7634	L	*260.1969*	130.6021	243.1703	122.0888			2
9							K	*147.1128*	74.0600	130.0863	65.5468			1

H1.0 (H1°): **LVTTGVLK(pr)QTK(pr)GVGASGSFR**

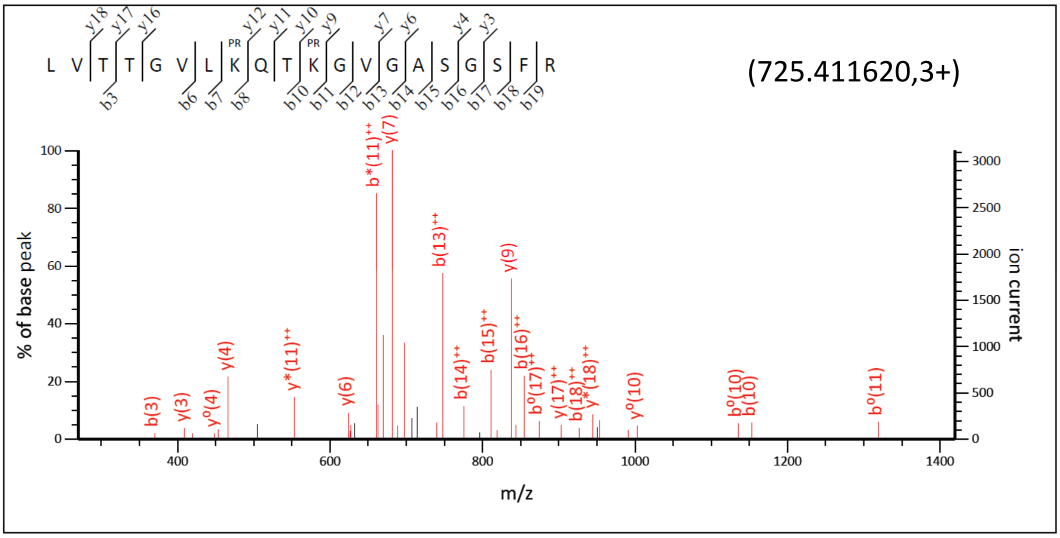

Monoisotopic mass of neutral peptide Mr(calc): 2173.2107
Fixed modifications: Carbamidomethyl (C),Propionyl (N-term) (apply to specified residues or termini only)
Variable modifications:
K8 : Propionyl (K)
K11 : Propionyl (K)
Ions Score: 64 **Expect:** 0.0001
Matches : 41/206 fragment ions using 40 most intense peaks (help)

#	b	b++	b*	b*++	b0	b0++	Seq.	y	y++	y*	y*++	y0	y0++	#
1	170.1176	85.5624					L							20
2	269.1860	135.0966					V	2005.1077	***1003.0575***	1988.0811	994.5442	1987.0971	994.0522	19
3	***370.2336***	185.6205			352.2231	176.6152	T	1906.0393	***953.5233***	1889.0127	945.0100	1888.0287	944.5180	18
4	471.2813	236.1443			453.2708	227.1390	T	1804.9916	***902.9994***	1787.9650	894.4862	1786.9810	893.9941	17
5	528.3028	264.6550			510.2922	255.6498	G	1703.9439	852.4756	1686.9173	843.9623	1685.9333	843.4703	16
6	***627.3712***	314.1892			609.3606	305.1840	V	1646.9224	823.9649	1629.8959	815.4516	1628.9119	814.9596	15
7	***740.4553***	370.7313			722.4447	361.7260	L	1547.8540	774.4306	1530.8275	765.9174	1529.8435	765.4254	14
8	924.5764	462.7919	907.5499	454.2786	906.5659	453.7866	K	1434.7700	717.8886	1417.7434	709.3753	1416.7594	708.8833	13
9	1052.6350	526.8212	1035.6085	518.3079	1034.6245	517.8159	Q	1250.6488	***625.8280***	1233.6222	617.3148	1232.6382	616.8227	12
10	***1153.6827***	577.3450	1136.6562	568.8317	1135.6721	568.3397	T	1122.5902	561.7987	1105.5637	553.2855	1104.5796	552.7935	11
11	1337.8039	***669.4056***	1320.7773	660.8923	1319.7933	660.4003	K	1021.5425	511.2749	1004.5160	502.7616	1003.5320	502.2696	10
12	1394.8253	***697.9163***	1377.7988	689.4030	1376.8148	688.9110	G	***837.4213***	***419.2143***	820.3948	410.7010	819.4108	410.2090	9
13	1493.8938	***747.4505***	1476.8672	738.9372	1475.8832	738.4452	V	780.3999	390.7036	763.3733	382.1903	762.3893	381.6983	8
14	1550.9152	***775.9613***	1533.8887	767.4480	1532.9047	766.9560	G	***681.3315***	341.1694	664.3049	332.6561	663.3209	332.1641	7
15	1621.9523	***811.4798***	1604.9258	802.9665	1603.9418	802.4745	A	***624.3100***	312.6586	607.2835	304.1454	606.2994	303.6534	6
16	1708.9844	***854.9958***	1691.9578	846.4825	1690.9738	845.9905	S	***553.2729***	277.1401	536.2463	268.6268	535.2623	268.1348	5
17	1766.0058	883.5066	1748.9793	874.9933	1747.9953	874.5013	G	***466.2409***	233.6241	449.2143	225.1108	448.2303	224.6188	4
18	1853.0379	***927.0226***	1836.0113	918.5093	1835.0273	918.0173	S	***409.2194***	205.1133	392.1928	196.6001	391.2088	196.1081	3
19	2000.1063	1000.5568	1983.0797	992.0435	1982.0957	991.5515	F	322.1874	161.5973	305.1608	153.0840			2
20							R	175.1190	88.0631	158.0924	79.5498			1

H1.0 (H1°): **LAK(pr)GDEPK(pr)R**

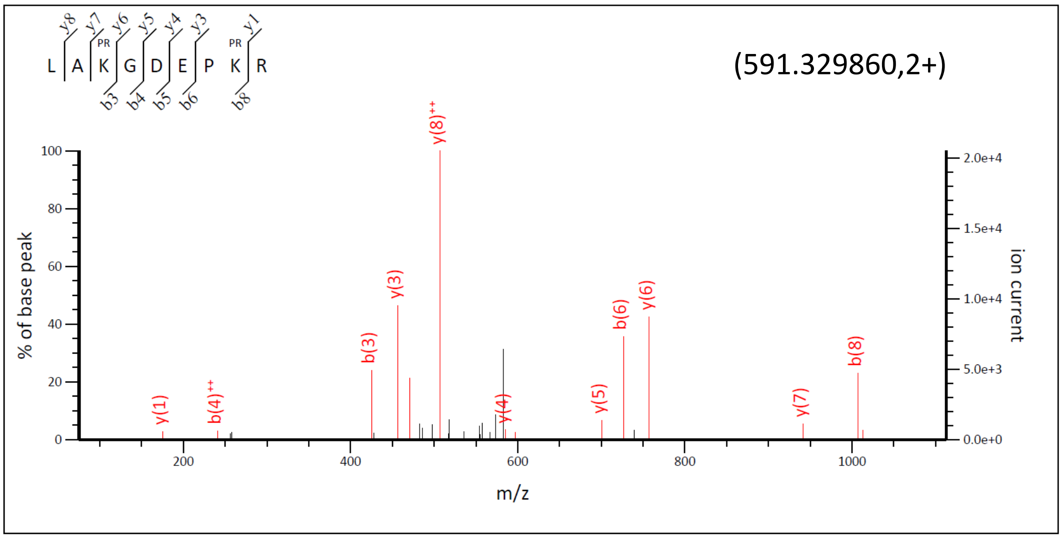

Monoisotopic mass of neutral peptide Mr(calc): 1180.6452
Fixed modifications: Carbamidomethyl (C),Propionyl (N-term) (apply to specified residues or termini only)
Variable modifications:
K3 : Propionyl (K)
K8 : Propionyl (K)
Ions Score: 65 **Expect:** 6.2e-05
Matches : 15/78 fragment ions using 18 most intense peaks (help)

#	b	b^{++}	b*	b^{*++}	b^0	b^{0++}	Seq.	y	y^{++}	y*	y^{*++}	y^0	y^{0++}	#
1	170.1176	85.5624					L							9
2	**241.1547**	121.0810					A	***1012.5422***	**506.7747**	995.5156	498.2615	994.5316	497.7694	8
3	**425.2758**	213.1416	408.2493	204.6283			K	***941.5051***	**471.2562**	924.4785	462.7429	923.4945	462.2509	7
4	482.2973	241.6523	465.2708	233.1390			G	***757.3839***	379.1956	740.3573	370.6823	739.3733	370.1903	6
5	**597.3243**	299.1658	580.2977	290.6525	579.3137	290.1605	D	***700.3624***	350.6849	683.3359	342.1716	682.3519	341.6796	5
6	**726.3668**	363.6871	709.3403	355.1738	708.3563	354.6818	E	***585.3355***	293.1714	568.3089	284.6581	567.3249	284.1661	4
7	823.4196	412.2134	806.3931	403.7002	805.4090	403.2082	P	***456.2929***	228.6501	439.2663	220.1368			3
8	**1007.5408**	504.2740	990.5142	495.7608	989.5302	495.2688	K	359.2401	180.1237	342.2136	171.6104			2
9							R	***175.1190***	88.0631	158.0924	79.5498			1

H1.0 (H1°): TENSTSAPAAK(pr)PK(pr)R

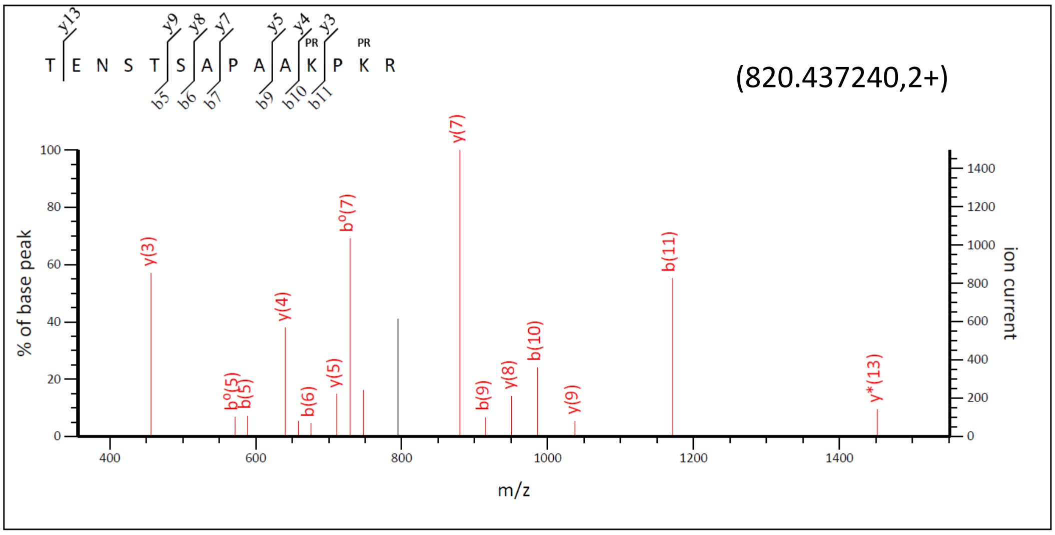

Monoisotopic mass of neutral peptide Mr(calc): 1624.8420
Fixed modifications: Carbamidomethyl (C),Propionyl (N-term) (apply to specified residues or termini only)
Variable modifications:
K11 : Propionyl (K)
K13 : Propionyl (K)
Ions Score: 74 **Expect:** 1.3e-05
Matches : 17/136 fragment ions using 17 most intense peaks (help)

#	b	b^{++}	b*	b^{*++}	b^0	b^{0++}	Seq.	y	y^{++}	y*	y^{*++}	y^0	y^{0++}	#
1	158.0812	79.5442			140.0706	70.5389	T							14
2	287.1238	144.0655			269.1132	135.0602	E	1468.7754	734.8914	1451.7489	726.3781	1450.7649	725.8861	13
3	401.1667	201.0870	384.1401	192.5737	383.1561	192.0817	N	1339.7328	670.3701	1322.7063	661.8568	1321.7223	661.3648	12
4	488.1987	244.6030	471.1722	236.0897	470.1882	235.5977	S	1225.6899	613.3486	1208.6634	604.8353	1207.6793	604.3433	11
5	***589.2464***	295.1268	572.2198	286.6136	571.2358	286.1216	T	1138.6579	569.8326	1121.6313	561.3193	1120.6473	560.8273	10
6	***676.2784***	338.6429	659.2519	330.1296	658.2679	329.6376	S	***1037.6102***	519.3087	1020.5837	510.7955	1019.5996	510.3035	9
7	***747.3155***	374.1614	730.2890	365.6481	729.3050	365.1561	A	***950.5782***	475.7927	933.5516	467.2795			8
8	844.3683	422.6878	827.3418	414.1745	826.3577	413.6825	P	***879.5411***	440.2742	862.5145	431.7609			7
9	***915.4054***	458.2063	898.3789	449.6931	897.3949	449.2011	A	782.4883	391.7478	765.4618	383.2345			6
10	***986.4425***	493.7249	969.4160	485.2116	968.4320	484.7196	A	***711.4512***	356.2292	694.4246	347.7160			5
11	***1170.5637***	585.7855	1153.5372	577.2722	1152.5531	576.7802	K	***640.4141***	320.7107	623.3875	312.1974			4
12	1267.6165	634.3119	1250.5899	625.7986	1249.6059	625.3066	P	***456.2929***	228.6501	439.2663	220.1368			3
13	***1451.7377***	726.3725	1434.7111	717.8592	1433.7271	717.3672	K	359.2401	180.1237	342.2136	171.6104			2
14							R	175.1190	88.0631	158.0924	79.5498			1

TS H1.7 (H1T2, HANP1): QTIQKPALDNAPSIQGK

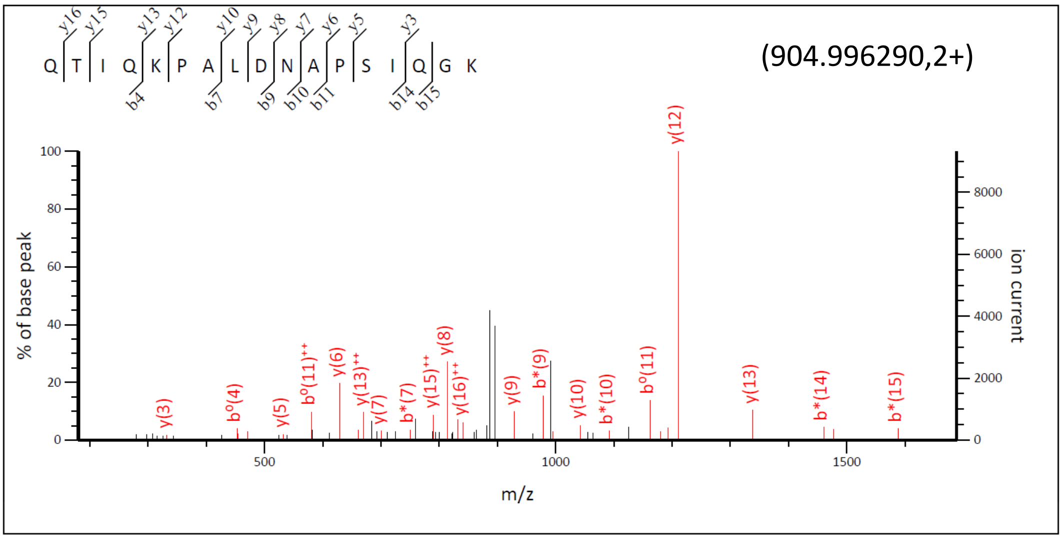

Monoisotopic mass of neutral peptide Mr(calc): 1807.9792
Fixed modifications: Carbamidomethyl (C) (apply to specified residues or termini only)
Ions Score: 63 **Expect:** 9.8e-06
Matches : 32/182 fragment ions using 41 most intense peaks (help)

#	b	b^{++}	b^{*}	b^{*++}	b^{0}	b^{0++}	Seq.	y	y^{++}	y^{*}	y^{*++}	y^{0}	y^{0++}	#
1	129.0659	65.0366	112.0393	56.5233			Q							17
2	230.1135	115.5604	213.0870	107.0471	212.1030	106.5551	T	1680.9279	**840.9676**	1663.9014	832.4543	1662.9173	831.9623	16
3	343.1976	172.1024	326.1710	163.5892	325.1870	163.0972	I	1579.8802	**790.4438**	1562.8537	781.9305	1561.8697	781.4385	15
4	**471.2562**	236.1317	454.2296	227.6185	453.2456	227.1264	Q	1466.7962	733.9017	1449.7696	725.3884	1448.7856	724.8964	14
5	599.3511	300.1792	582.3246	291.6659	581.3406	291.1739	K	***1338.7376***	**669.8724**	1321.7110	661.3592	1320.7270	660.8672	13
6	696.4039	348.7056	679.3774	340.1923	678.3933	339.7003	P	***1210.6426***	605.8250	1193.6161	597.3117	1192.6321	596.8197	12
7	767.4410	384.2241	750.4145	375.7109	749.4305	375.2189	A	1113.5899	557.2986	1096.5633	548.7853	1095.5793	548.2933	11
8	880.5251	440.7662	863.4985	432.2529	862.5145	431.7609	L	***1042.5528***	521.7800	1025.5262	513.2667	1024.5422	512.7747	10
9	**995.5520**	498.2796	978.5255	489.7664	977.5415	489.2744	D	***929.4687***	465.2380	912.4421	456.7247	911.4581	456.2327	9
10	1109.5950	555.3011	1092.5684	546.7878	1091.5844	546.2958	N	***814.4417***	407.7245	797.4152	399.2112	796.4312	398.7192	8
11	**1180.6321**	590.8197	1163.6055	582.3064	1162.6215	581.8144	A	***700.3988***	350.7030	683.3723	342.1898	682.3883	341.6978	7
12	1277.6848	639.3461	1260.6583	630.8328	1259.6743	630.3408	P	***629.3617***	315.1845	612.3352	306.6712	611.3511	306.1792	6
13	1364.7169	682.8621	1347.6903	674.3488	1346.7063	673.8568	S	***532.3089***	266.6581	515.2824	258.1448	514.2984	257.6528	5
14	**1477.8009**	739.4041	1460.7744	730.8908	1459.7904	730.3988	I	445.2769	223.1421	428.2504	214.6288			4
15	1605.8595	803.4334	1588.8329	794.9201	1587.8489	794.4281	Q	***332.1928***	166.6001	315.1663	158.0868			3
16	1662.8810	831.9441	1645.8544	823.4308	1644.8704	822.9388	G	204.1343	102.5708	187.1077	94.0575			2
17							K	147.1128	74.0600	130.0863	65.5468			1

TS H1.7 (H1T2, HANP1): **VSGSDAAGYFR**

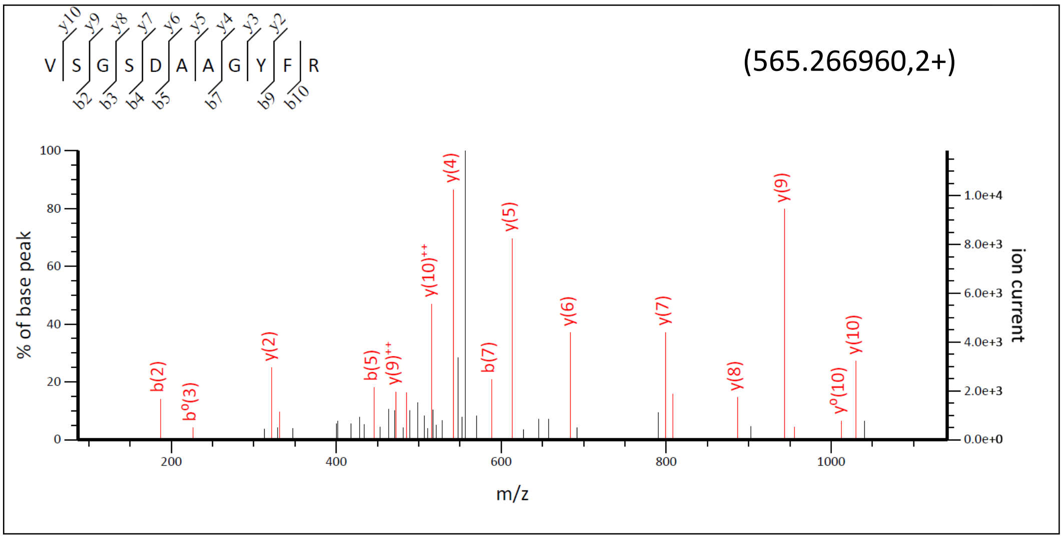

Monoisotopic mass of neutral peptide Mr(calc): 1128.5200
Fixed modifications: Carbamidomethyl (C) (apply to specified residues or termini only)
Ions Score: 89 **Expect:** 2.3e-08
Matches : 19/86 fragment ions using 24 most intense peaks (help)

#	b	b⁺⁺	b⁰	b⁰⁺⁺	Seq.	y	y⁺⁺	y*	y*⁺⁺	y⁰	y⁰⁺⁺	#
1	100.0757	50.5415			V							11
2	**187.1077**	94.0575	169.0972	85.0522	S	***1030.4589***	**515.7331**	1013.4323	507.2198	1012.4483	506.7278	10
3	244.1292	122.5682	226.1186	113.5629	G	***943.4268***	**472.2170**	926.4003	463.7038	925.4163	463.2118	9
4	**331.1612**	166.0842	313.1506	157.0790	S	***886.4054***	443.7063	869.3788	435.1930	868.3948	434.7010	8
5	**446.1882**	223.5977	428.1776	214.5924	D	***799.3733***	400.1903	782.3468	391.6770	781.3628	391.1850	7
6	517.2253	259.1163	499.2147	250.1110	A	***684.3464***	342.6768	667.3198	334.1636			6
7	**588.2624**	294.6348	570.2518	285.6295	A	***613.3093***	307.1583	596.2827	298.6450			5
8	645.2838	323.1456	627.2733	314.1403	G	***542.2722***	271.6397	525.2456	263.1264			4
9	**808.3472**	404.6772	790.3366	395.6719	Y	***485.2507***	243.1290	468.2241	234.6157			3
10	**955.4156**	478.2114	937.4050	469.2061	F	***322.1874***	161.5973	305.1608	153.0840			2
11					R	175.1190	88.0631	158.0924	79.5498			1

TS H1.7 (H1T2, HANP1): AIAGHQHLTLDALK

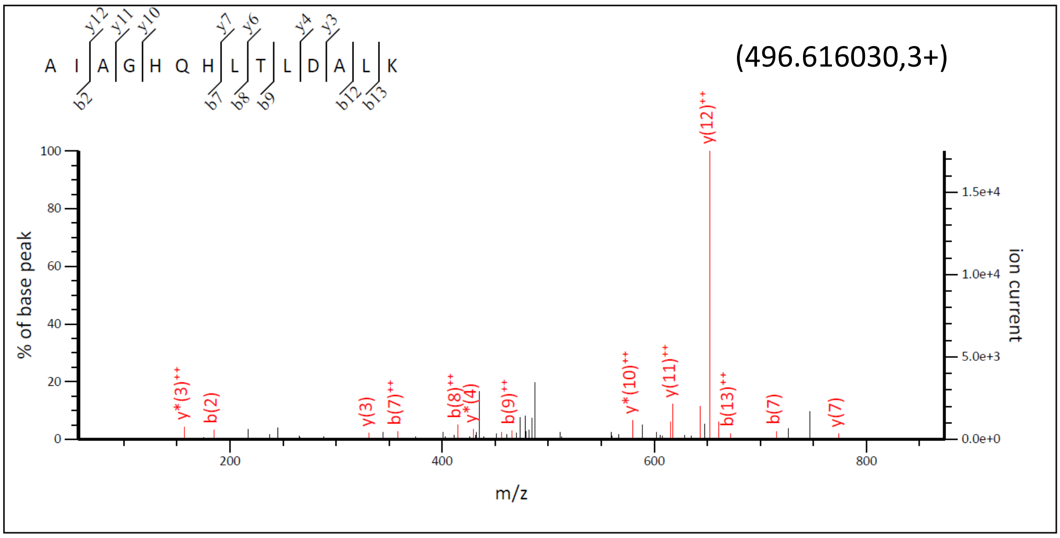

Monoisotopic mass of neutral peptide Mr(calc): 1486.8256
Fixed modifications: Carbamidomethyl (C) (apply to specified residues or termini only)
Ions Score: 41 **Expect:** 0.011
Matches : 25/124 fragment ions using 35 most intense peaks (help)

#	b	b++	b*	b*++	b0	b0++	Seq.	y	y++	y*	y*++	y0	y0++	#
1	72.0444	36.5258					A							14
2	185.1285	93.0679					I	1416.7958	708.9015	1399.7692	700.3883	1398.7852	699.8962	13
3	256.1656	128.5864					A	1303.7117	652.3595	1286.6852	643.8462	1285.7011	643.3542	12
4	313.1870	157.0972					G	1232.6746	616.8409	1215.6480	608.3277	1214.6640	607.8357	11
5	450.2459	225.6266					H	1175.6531	588.3302	1158.6266	579.8169	1157.6426	579.3249	10
6	578.3045	289.6559	561.2780	281.1426			Q	1038.5942	519.8007	1021.5677	511.2875	1020.5837	510.7955	9
7	715.3634	358.1854	698.3369	349.6721			H	910.5356	455.7715	893.5091	447.2582	892.5251	446.7662	8
8	828.4475	414.7274	811.4209	406.2141			L	773.4767	387.2420	756.4502	378.7287	755.4662	378.2367	7
9	929.4952	465.2512	912.4686	456.7380	911.4846	456.2459	T	660.3927	330.7000	643.3661	322.1867	642.3821	321.6947	6
10	1042.5792	521.7933	1025.5527	513.2800	1024.5687	512.7880	L	559.3450	280.1761	542.3184	271.6629	541.3344	271.1708	5
11	1157.6062	579.3067	1140.5796	570.7935	1139.5956	570.3014	D	446.2609	223.6341	429.2344	215.1208	428.2504	214.6288	4
12	1228.6433	614.8253	1211.6167	606.3120	1210.6327	605.8200	A	331.2340	166.1206	314.2074	157.6074			3
13	1341.7274	671.3673	1324.7008	662.8540	1323.7168	662.3620	L	260.1969	130.6021	243.1703	122.0888			2
14							K	147.1128	74.0600	130.0863	65.5468			1

TS H1.7 (H1T2, HANP1): **ELGNAGYEVR**

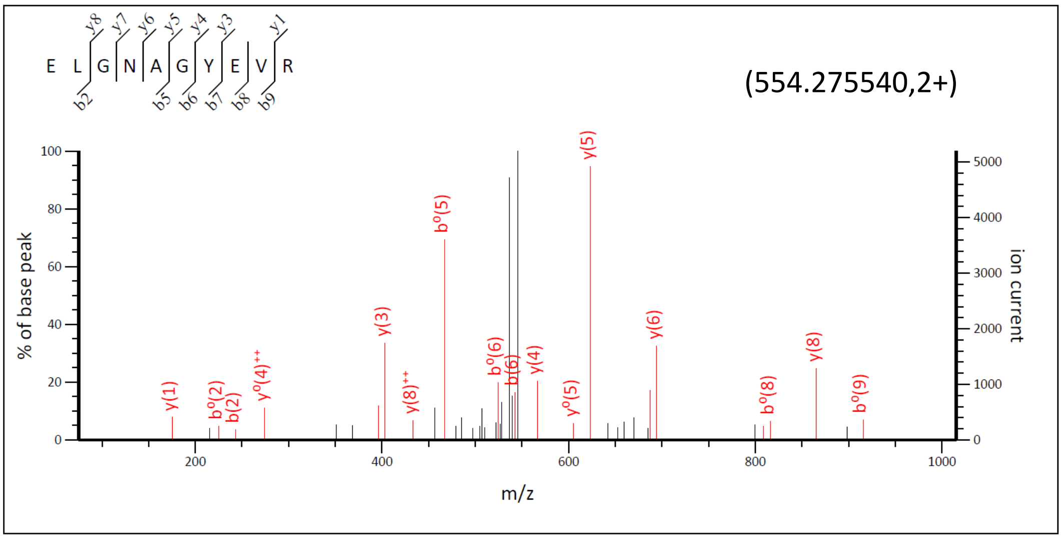

Monoisotopic mass of neutral peptide Mr(calc): 1106.5356
Fixed modifications: Carbamidomethyl (C) (apply to specified residues or termini only)
Ions Score: 64 **Expect:** 1.5e-05
Matches : 24/98 fragment ions using 31 most intense peaks (help)

#	b	b++	b*	b*++	b0	b0++	Seq.	y	y++	y*	y*++	y0	y0++	#
1	130.0499	65.5286			112.0393	56.5233	E							10
2	**243.1339**	122.0706			225.1234	113.0653	L	978.5003	489.7538	961.4738	481.2405	960.4898	480.7485	9
3	300.1554	150.5813			282.1448	141.5761	G	***865.4163***	433.2118	848.3897	424.6985	847.4057	424.2065	8
4	414.1983	207.6028	397.1718	199.0895	396.1878	198.5975	N	***808.3948***	404.7010	791.3682	396.1878	790.3842	395.6958	7
5	485.2354	**243.1214**	468.2089	234.6081	467.2249	234.1161	A	***694.3519***	347.6796	677.3253	339.1663	676.3413	338.6743	6
6	**542.2569**	271.6321	525.2304	263.1188	524.2463	262.6268	G	***623.3148***	312.1610	606.2882	303.6477	605.3042	303.1557	5
7	705.3202	353.1638	688.2937	344.6505	687.3097	344.1585	Y	***566.2933***	283.6503	549.2667	275.1370	548.2827	274.6450	4
8	834.3628	417.6851	817.3363	409.1718	816.3523	408.6798	E	***403.2300***	202.1186	386.2034	193.6053	385.2194	193.1133	3
9	933.4312	**467.2193**	916.4047	458.7060	915.4207	458.2140	V	***274.1874***	137.5973	257.1608	129.0840			2
10							R	***175.1190***	88.0631	158.0924	79.5498			1

TS H1.7 (H1T2, HANP1): **AEAVQPSGESQGAELTIQIQQPAER**

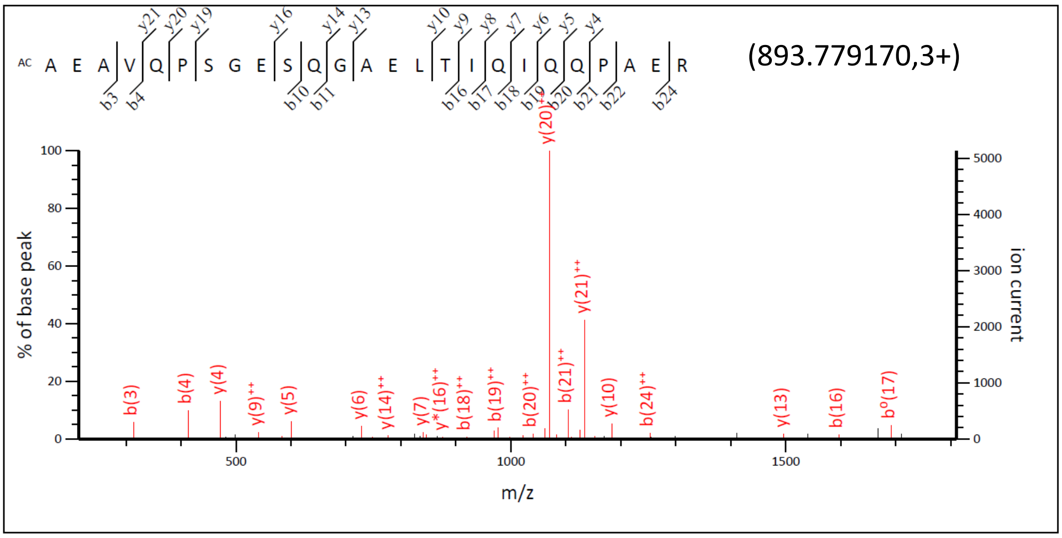

Monoisotopic mass of neutral peptide Mr(calc): 2678.3147
Fixed modifications: Carbamidomethyl (C),Propionyl (N-term) (apply to specified residues or termini only)
Variable modifications:
N-term : Acetyl (Protein N-term)
Ions Score: 60 **Expect:** 8e-05
Matches : 47/276 fragment ions using 46 most intense peaks (help)

#	b	b^{++}	b*	b^{*++}	b^0	b^{0++}	Seq.	y	y^{++}	y*	y^{*++}	y^0	y^{0++}	#
1	114.0550	57.5311					A							25
2	243.0975	122.0524			225.0870	113.0471	E	2566.2744	1283.6408	2549.2478	1275.1275	2548.2638	1274.6355	24
3	***314.1347***	157.5710			296.1241	148.5657	A	2437.2318	1219.1195	2420.2052	1210.6062	2419.2212	1210.1142	23
4	***413.2031***	207.1052			395.1925	198.0999	V	2366.1946	***1183.6010***	2349.1681	1175.0877	2348.1841	1174.5957	22
5	***541.2617***	271.1345	524.2351	262.6212	523.2511	262.1292	Q	2267.1262	***1134.0668***	2250.0997	1125.5535	2249.1157	1125.0615	21
6	638.3144	319.6608	621.2879	311.1476	620.3039	310.6556	P	2139.0677	***1070.0375***	2122.0411	1061.5242	2121.0571	1061.0322	20
7	725.3464	363.1769	708.3199	354.6636	707.3359	354.1716	S	2042.0149	***1021.5111***	2024.9883	1012.9978	2024.0043	1012.5058	19
8	782.3679	391.6876	765.3414	383.1743	764.3573	382.6823	G	1954.9829	977.9951	1937.9563	969.4818	1936.9723	968.9898	18
9	911.4105	456.2089	894.3840	447.6956	893.3999	447.2036	E	1897.9614	949.4843	1880.9348	940.9711	1879.9508	940.4791	17
10	***998.4425***	499.7249	981.4160	491.2116	980.4320	490.7196	S	1768.9188	884.9630	1751.8923	876.4498	1750.9082	875.9578	16
11	1126.5011	563.7542	1109.4746	555.2409	1108.4905	554.7489	Q	1681.8868	***841.4470***	1664.8602	832.9338	1663.8762	832.4417	15
12	***1183.5226***	592.2649	1166.4960	583.7517	1165.5120	583.2596	G	1553.8282	***777.4177***	1536.8016	768.9045	1535.8176	768.4125	14
13	1254.5597	627.7835	1237.5331	619.2702	1236.5491	618.7782	A	***1496.8067***	***748.9070***	1479.7802	740.3937	1478.7962	739.9017	13
14	1383.6023	692.3048	1366.5757	683.7915	1365.5917	683.2995	E	1425.7696	713.3884	1408.7431	704.8752	1407.7591	704.3832	12
15	***1496.6863***	***748.8468***	1479.6598	740.3335	1478.6758	739.8415	L	1296.7270	648.8672	1279.7005	640.3539	1278.7165	639.8619	11
16	***1597.7340***	799.3706	1580.7075	790.8574	1579.7235	790.3654	T	***1183.6430***	592.3251	1166.6164	583.8118	1165.6324	583.3198	10
17	1710.8181	855.9127	1693.7915	847.3994	1692.8075	846.9074	I	***1082.5953***	***541.8013***	1065.5687	533.2880	1064.5847	532.7960	9
18	1838.8767	***919.9420***	1821.8501	911.4287	1820.8661	910.9367	Q	***969.5112***	485.2592	952.4847	476.7460	951.5007	476.2540	8
19	1951.9607	***976.4840***	1934.9342	967.9707	1933.9502	967.4787	I	***841.4526***	421.2300	824.4261	412.7167	823.4421	412.2247	7
20	2080.0193	***1040.5133***	2062.9928	1032.0000	2062.0087	1031.5080	Q	***728.3686***	364.6879	711.3420	356.1747	710.3580	355.6826	6
21	2208.0779	***1104.5426***	2191.0513	1096.0293	2190.0673	1095.5373	Q	***600.3100***	300.6586	583.2835	292.1454	582.2994	291.6534	5
22	2305.1306	***1153.0690***	2288.1041	1144.5557	2287.1201	1144.0637	P	***472.2514***	236.6293	455.2249	228.1161	454.2409	227.6241	4
23	2376.1678	1188.5875	2359.1412	1180.0742	2358.1572	1179.5822	A	375.1987	188.1030	358.1721	179.5897	357.1881	179.0977	3
24	2505.2104	***1253.1088***	2488.1838	1244.5955	2487.1998	1244.1035	E	304.1615	152.5844	287.1350	144.0711	286.1510	143.5791	2
25							R	175.1190	88.0631	158.0924	79.5498			1

TS H1.7 (H1T2, HANP1): SSAKSSAKSSTR

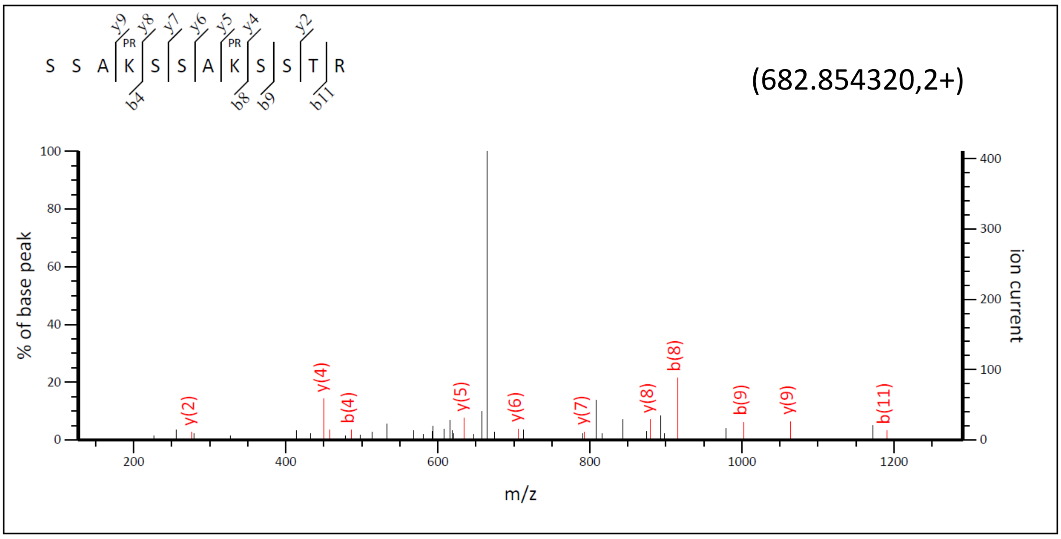

Monoisotopic mass of neutral peptide Mr(calc): 1363.6943
Fixed modifications: Carbamidomethyl (C),Propionyl (N-term) (apply to specified residues or termini only)
Variable modifications:
K4 : Propionyl (K)
K8 : Propionyl (K)
Ions Score: 33 **Expect:** 0.026
Matches : 12/124 fragment ions using 31 most intense peaks (help)

#	b	b^{++}	b*	b^{*++}	b^0	b^{0++}	Seq.	y	y^{++}	y*	y^{*++}	y^0	y^{0++}	#
1	144.0655	72.5364			126.0550	63.5311	S							12
2	231.0975	116.0524			213.0870	107.0471	S	1221.6434	611.3253	1204.6168	602.8120	1203.6328	602.3200	11
3	302.1347	151.5710			284.1241	142.5657	A	1134.6113	567.8093	1117.5848	559.2960	1116.6008	558.8040	10
4	*486.2558*	243.6316	469.2293	235.1183	468.2453	234.6263	K	*1063.5742*	532.2907	1046.5477	523.7775	1045.5636	523.2855	9
5	573.2879	287.1476	556.2613	278.6343	555.2773	278.1423	S	*879.4530*	440.2302	862.4265	431.7169	861.4425	431.2249	8
6	660.3199	330.6636	643.2933	322.1503	642.3093	321.6583	S	*792.4210*	396.7141	775.3945	388.2009	774.4104	387.7089	7
7	731.3570	366.1821	714.3305	357.6689	713.3464	357.1769	A	*705.3890*	353.1981	688.3624	344.6849	687.3784	344.1928	6
8	*915.4782*	458.2427	898.4516	449.7295	897.4676	449.2374	K	*634.3519*	317.6796	617.3253	309.1663	616.3413	308.6743	5
9	*1002.5102*	501.7587	985.4837	493.2455	984.4996	492.7535	S	*450.2307*	225.6190	433.2041	217.1057	432.2201	216.6137	4
10	1089.5422	545.2748	1072.5157	536.7615	1071.5317	536.2695	S	363.1987	182.1030	346.1721	173.5897	345.1881	173.0977	3
11	*1190.5899*	595.7986	1173.5634	587.2853	1172.5794	586.7933	T	*276.1666*	138.5870	259.1401	130.0737	258.1561	129.5817	2
12							R	175.1190	88.0631	158.0924	79.5498			1
